# Highly efficient chromatin conformation capture with post-enrichment in single cells by HiChew

**DOI:** 10.1186/s13059-026-04059-1

**Published:** 2026-04-27

**Authors:** Zhichao Chen, Yeming Xie, Chen Zhang, Chen Tan, Fengying Ruan, Wenfang Chen, Meng Luo, Mei Guo, Zhe Xie, Huimin Cao, Meifang Tang, Xiuqing Zhang, Ying Zhang, Chong Tang

**Affiliations:** 1 College of Life Sciences, University of Chinese Academy of Sciences, Beijing, 100049 China; 2 BGI Genomics, BGI-Shenzhen, Shenzhen, 518083 China; 3 BGI Research, Shenzhen, 518083 China; 4 NHC Key Laboratory of Male Reproduction and Genetics, Guangzhou, 510600 China; 5 Human Sperm Bank of Guangdong Province, Guangdong Provincial Reproductive Science Institute, Guangdong Provincial Fertility Hospital, Guangzhou, 510600 China

**Keywords:** Chromatin conformation capture, Single-cell Hi-C, HiChew, Post-enrichment method, DNA methylation, High-throughput sequencing, 3D genome structure, Chromatin interactions, Valid pair ratio, Nuclear architecture

## Abstract

We present HiChew, a novel chromatin conformation capture method combining efficient sticky-end ligation with post-PCR methylation-based enrichment. HiChew achieves approximately 50% valid pair ratios compared to 8% for unenriched methods, while maintaining high sensitivity. Its single-cell implementation, snHiChew, achieves 45–50% valid pair ratios, and enables 5–10 kb resolution mapping with 70–80% bin coverage. Comparative analyses show strong concordance with conventional Hi-C for chromatin compartments, TADs, and loops. By combining scalability, cost-effectiveness and data quality, HiChew provides a powerful platform for advancing studies of 3D genome architecture analysis.

**Supplementary Information:**

The online version contains supplementary material available at 10.1186/s13059-026-04059-1.

## Background

Chromatin conformation capture methods are useful tools for exploring the three-dimensional structure of chromatin in cells. These methods enable researchers to observe how the genome is arranged in space, which is essential for comprehending gene expression and regulation [1].

Chromatin conformation capture [2] is a technique that involves crosslinking genomic DNA, digesting it with a restriction enzyme, end repair to incorporate the biotin, and then ligating the resulting fragments. To enrich the ligated products, we usually adopt the streptavidin enrichment method, which helps to generate more valid pairs. In conventional Hi-C, the ligation occurs between proximal fragments, providing information about the spatial proximity of different regions of the genome. We then sequence the resulting library to identify the interactions between different regions of the genome.

However, because the genome structure can vary greatly between different cells, the conformation observed is actually an average of what is seen in various cells. In order to observe the unique structures of individual cells and avoid any interference from neighboring cells, researchers have developed single-cell Hi-C [3]. This method offers the advantage of a more precise observation of the dynamic nature of chromatin structures. There are two main styles of methods in the single-cell Hi-C field: enrichment methods and non-enrichment methods.

In the initial stages, researchers developed the first-generation single-cell Hi-C protocol (Fig. S1a), which was similar to conventional Hi-C but optimized for minimal input DNA [3–6]. However, end-repaired blunt ends have lower ligation efficiency than sticky ends. Additionally, biotin enrichment also leads to DNA loss during the immunoprecipitation process.

Lin et. al. developed a new method, called DLO Hi-C, to solve this problem [7, 8]. The method involves inserting two linkers with a specific sequence between neighboring DNA fragments. These linkers contain a specific sequence with digestion recognition sites at the junction position. After successful ligation, MDA(multiple strand displacement amplification) can be performed, and the ligated junctions can be enriched by specific digestion with recognition sites on the linkers. This technique relies on the adapter's specific sequence instead of biotins for enrichment, which reduces the loss of enrichment in minimal input DNA. MDA can generate ample DNA for downstream enrichment, eliminating the need for biotin purification and amplifying the DNA before enrichment. However, the successful ligation rate may decrease because this step requires two linker ligations in the junction position, meaning that the fragments must be ligated three times.

While enrichment-based methods have their merits, alternative approaches such as Dip-C [9], scCARE-seq [10], HIRES [11] offer direct ligation of neighboring fragments without the need for end repair(Fig. S1b). This approach facilitates more efficient sticky-end ligation compared to methods using biotin-incorporated blunt ends. However, it's crucial to note that full genome sequencing in these methods may result in a significant proportion of reads being discarded. For instance, Dip-C requires 10–47 Gb sequencing per cell to yield approximately 1 M contacts [9], as the ligation scar represents only a small fraction of the genome. This inefficiency could potentially impact the detection of rare DNA conformations.

When carrying out single-cell chromatin conformation capture (3C) experiments, the selection of methods merits careful consideration. In order to cut down on sequencing costs and detect rare contacts more effectively, a method that can manage large-scale cell numbers and ultra-deep sequencing per cell is required. Our approach, HiChew (Highly efficient chromatin conformation capture with post-enrichment), is notable for its high efficiency. HiChew is unique as it circumvents the need for pre-enrichment prior to amplification. We employ a high-efficiency sticky-end ligation method, akin to Dip-C and HIRES, which reduces the steps required before amplification. Upon amplification, we mark the ligation scar motifs with a corresponding methyltransferase and enrich the ligation scars using a methylation antibody. In summary, our approach has demonstrated improved efficiency for single-cell 3 C to date, enhancing not only single-cell 3 C but also simplifying conventional Hi-C in common labs.

## Results

### Principle of the HiChew design

**Fig. 1:**
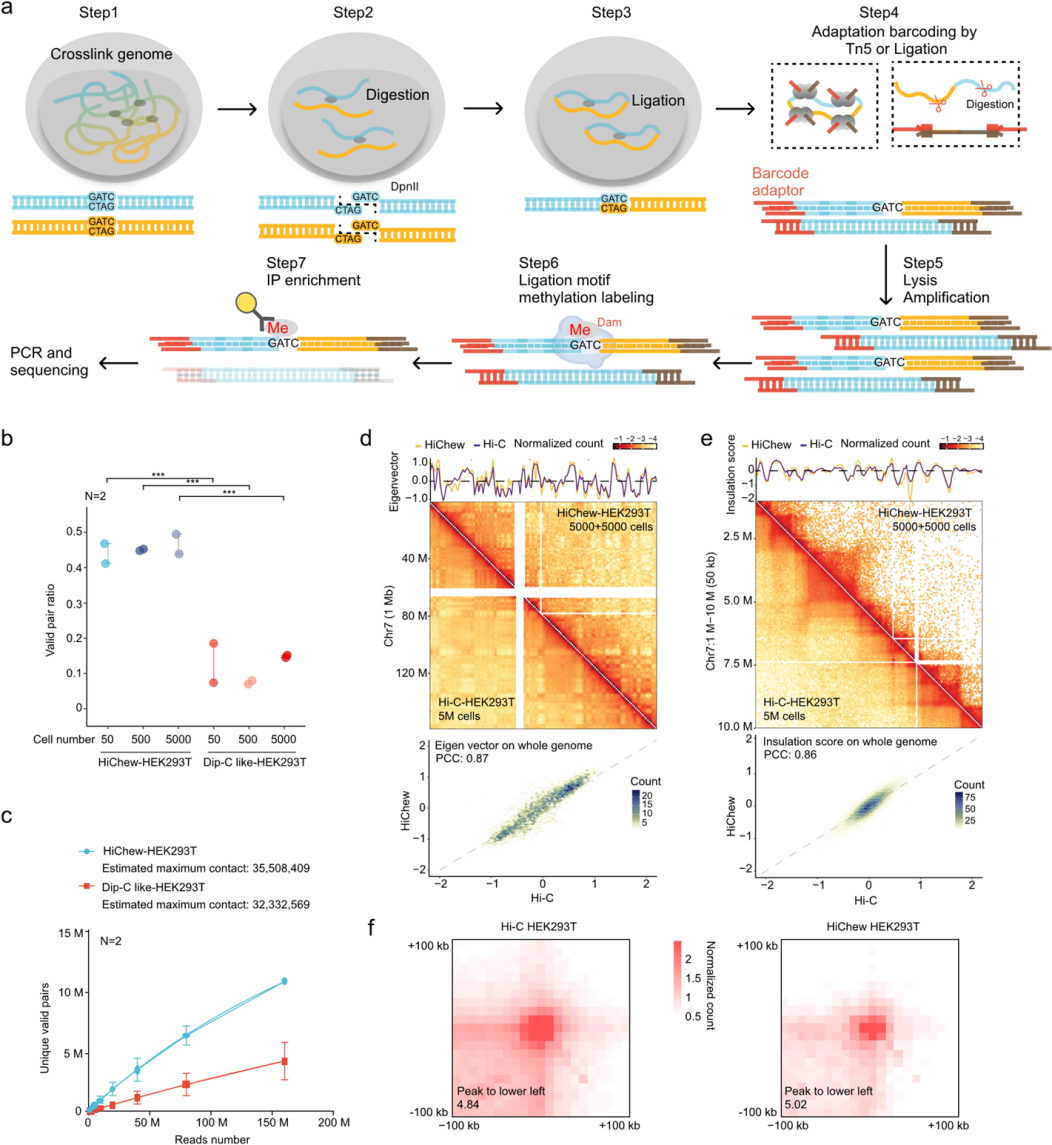
Overview and validation of the HiChew method for chromatin interaction analysis. **a** Schematic representation of the HiChew protocol. The workflow consists of seven key steps: genome crosslinking, DNA digestion, proximal ligation, barcode adaptation, DNA amplification, methylation labeling, and IP enrichment followed by PCR and sequencing. **b** Comparison of valid pair ratios between HiChew and Dip-C like methods across different cell input numbers (50, 500, and 5000 cells), demonstrated that HiChew is more efficient than Dip-C like method (*N* = 2, *** indicates *P* < 0.001). *P* values are from two-sided two-sample t-tests. The error bar represents the standard error (SE). **c** Saturation analysis comparing HiChew and Dip-C methods using 50-cell samples. The line graph shows unique valid pairs versus total reads number, indicating the library complexity. The error bar represents the standard deviation (SD). **d** and (**e**) Comparison and Analysis of Chromatin Organization at Chromosome 7. Top: Contact matrices comparing Hi-C (5 million cells) and HiChew (two biological replicates of 5,000 cells) methods at chromosome 7. The matrices are displayed at 500 kb resolution, with corresponding eigenvalue tracks above (left). Additionally, a detailed view of a chr7: 40 M-50 M region is shown at 50 kb resolution, with corresponding insulation score tracks above (right). Bottom: Correlation plots demonstrating high concordance between Hi-C and HiChew methods, including an eigenvalue correlation plot for overall chromatin organization (left) and an insulation score correlation plot for local chromatin organization (right). **f** Aggregate peak analysis (APA) plots comparing loop strength between Hi-C and HiChew methods, with enrichment scores to the lower left shown below each plot

The HiChew protocol consists of four essential steps prior to PCR amplification. Initially, we employ formaldehyde to crosslink DNA strands and proteins (Fig. 1a, step 1). Subsequently, we utilize DpnII to cleave the DNA at GATC motifs (Fig. 1a, step 2). Following this, we implement proximal ligation to join nearby digested DNA ends, resulting in a GATC ligation scar (Fig. 1a, step 3). After cell lysis, we proceed with DNA library construction, selecting either an adapter ligation-based or Tn5-based method, contingent upon DNA quantity and experimental requirements (Fig. 1a, step 4).

Following PCR amplification, we employ dam methyltransferase to label the ligation scar with m6A methylation on the GATC motif. Subsequently, we implement an immunoprecipitation (IP) protocol to selectively enrich the ligation scars using m6A antibody pull-down (Fig. 1a, steps 6–7). This post-PCR enrichment strategy enhances the efficiency of our chromatin conformation capture process.

HiChew addresses challenges in low cell quantity or single-cell Hi-C biotin enrichment by streamlining the protocol. It focuses on digestion and direct ligation, eliminating steps such as end repairing, biotin incorporation, blunt end ligation, and biotin enrichment (Fig. S1a). This approach enhances ligation efficiency and minimizes pre-PCR amplification steps, reducing potential cell or DNA loss (Fig. S1c). The method's post-PCR enrichment process allows for enrichment using abundant DNA, mitigating drawbacks associated with biotin enrichment prior to PCR. Additionally, HiChew resolves the inefficiency of Dip-C (Fig. S1b), which requires whole genome sequencing and consumes a significant portion of the sequencing budget. HiChew generates a high proportion of valid pair information, optimizing resource utilization and data quality.

### The performance of HiChew is similar to Hi-C in low-input bulk sample

We compared two chromatin conformation capture methods: unenriched Dip-C and HiChew. We conducted experiments using HEK293T cells at three quantities (5,000, 500, and 50 cells) in triplicate, with sequencing depths of 100–200 million paired-end reads (Table S1). Initial quality assessment revealed that both methods achieved similar performance metrics, including cis–trans ratios and distance decay curves (Fig. S1e-f). However, Dip-C exhibited higher rates of unmapped and invalid pairs (dangling end pais and religation pairs, Fig. S1d), which was expected given its unenriched methodology. The quality control definitions follow HiC-Pro [12].

**Fig. 2:**
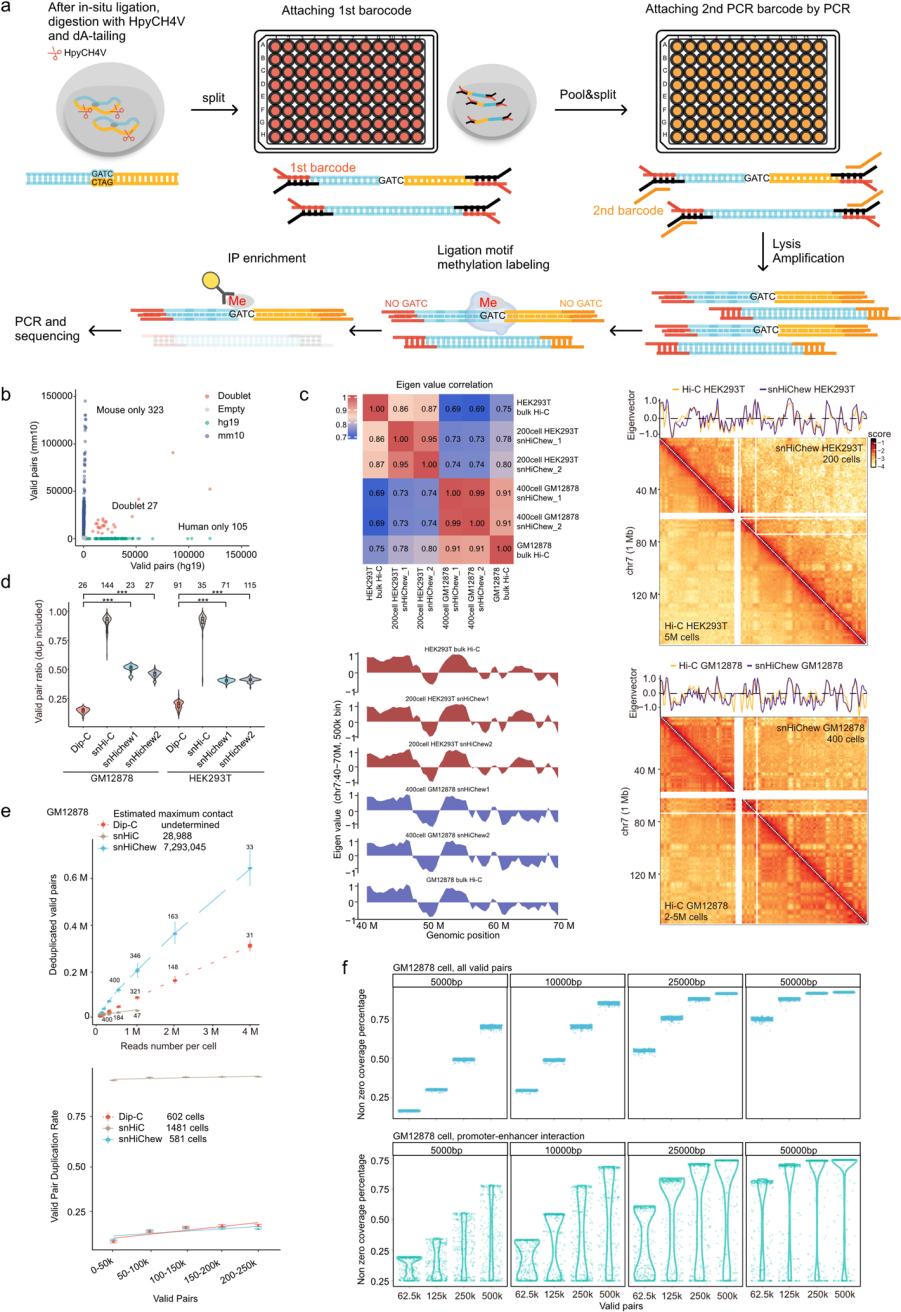
Development and validation of the snHiChew methodology. **a** Systematic overview of the snHiChew protocol, encompassing sequential steps: in-situ ligation and digestion, followed by dual-stage barcode incorporation. Initial barcode integration occurs in plate format (indicated by red markers), succeeded by sample pooling and secondary barcode addition via PCR amplification (denoted by orange markers). The protocol proceeds with cellular lysis, DNA amplification, methylation-specific labeling of ligation junctions, immunoprecipitation enrichment, and culminates in PCR amplification and sequencing analysis. **b** Scatter plots showing the collision level in the snHiChew library by unique valid pairs identified from the mm10 (mouse) and hg19 (human) genomic alignment, revealing discrete populations: mouse-specific (blue), human-specific (cyan), and doublet cells (red). **c** Comparative correlation assessment and matrix evaluation of snHiChew methodologies in HEK293T and GM12878 cell lines. Left top: Pearson’s correlation between the aggregated snHiChew replicates and the bulk in situ Hi-C data. Left bottom: Comparison of A/B compartments calling between snHiChew (pseudobulk) and in situ Hi-C. Right panel: Comparative visualization of Hi-C contact matrices between conventional Hi-C and illustrating maintained chromatin architectural features, with corresponding eigenvalue tracks displayed above. **d** Violin plot of valid pairs over successfully mapped reads (reported pair) of Dip-C, snHi-C, and two replicates of snHiChew across GM12878 and HEK293T. Cells of similar total reads are used for evaluation. The cell number for each violin plot is indicated at the top. Two-sided Wilcoxon signed-rank test was used, *** indicates *P* < 0.001. **e** The sequencing saturation curve is displayed to evaluate unique valid pair metrics relative to sequencing depth across Dip-C, snHi-C, and snHiChew. The x-axis represents the sequencing depth threshold for each cell, and the y-axis represents the deduped valid pairs from the corresponding sequencing depth. The cell number for each read threshold is indicated at the top. The error bar represents the standard deviation (SD). The bottom plot showed the valid pair duplication rate to the generated valid pairs. **f** Dot plots demonstrating the percentage of non-zero coverage bin across multiple bin resolutions (5000, 10,000, 25,000, and 50,000 bp) under different unique valid pairs thresholds (62.5 k, 125.0 k, 250.0 k, and 500.0 k valid pairs per cell) for 287 valid cells in snHiChew GM12878 library

We quantified the efficiency improvements of HiChew compared to Dip-C using the benchmark valid pair ratio (valid pairs/reported pairs). This metric is particularly useful because it normalizes by reported pairs, which remains stable across various sequencing depths (Fig. S1g). HiChew achieved a valid pair ratio of approximately 50% (*N* = 2) (Fig. 1b). Though this was lower than conventional Hi-C's 60 ~ 90%, HiChew significantly outperformed the unenriched Dip-C method by fourfold, as Dip-C achieved only an 8–15% valid pair ratio (Fig. 1b). These results demonstrate HiChew's superior efficiency in chromatin conformation capture, producing approximately four times more valid pairs than Dip-C. Additional validation with GM12878 cells and tissues confirmed consistent valid pair ratios of 40–50% (Figs. 2d, 5a).

Although HiChew demonstrates improved capture efficiency, we are investigating whether this increased efficiency might potentially affect the sensitivity of these technologies, leading to a loss of contact information. A notable challenge in single-cell/low-input Hi-C is that while biotin purification enhances the proportion of valid pairs and reduces sequencing costs, it also results in substantial DNA loss and diminished signal strength. This limitation likely explains the recent trend toward Dip-C in single-cell studies.

To evaluate whether HiChew maintains contact information integrity during processing, we conducted a comprehensive analysis of deduplicated valid pairs across multiple sequencing depths to assess library complexity. Our analysis demonstrates that HiChew consistently produces 3–4 times more unique valid contacts compared to the unenriched Dip-C method (Fig. 1c). Our sequencing saturation analysis indicates that HiChew, utilizing only 50 cells, generates a 10% higher estimated maximum contact (35 M) compared to Dip-C's 32 M with the same cell input (Fig. 1c, *N* = 2), suggesting comparable theoretical maximum library complexity between HiChew and Dip-C.

We next evaluated how accurately the valid pairs represented chromatin conformation. Using 5000 cells, HiChew performed similarly to Hi-C across multiple resolution levels. When comparing HiChew (5000 + 5000 cells) to standard Hi-C (5 M cells), we observed an eigenvalue correlation of 0.87 and an insulation score correlation of 0.86 (Fig. 1d,e). Loop detection analysis showed comparable enrichment signals in APA plots between the two methods (peak to lower left: 4.84 vs 5.02) (Fig. 1f). While the correlation in AB compartment scores and TAD insulation scores predictably decreased with fewer cells (Fig. S2b,c), HiChew still surpassed Dip-C's performance thanks to its higher contact yield (Fig. S2a-c).

Our evaluation of inter-batch reproducibility involved analyzing eigenvalue correlations across varying cell inputs. The results demonstrated HiChew's exceptional consistency, with batch correlations achieving eigenvalue correlations of 0.96, 0.98 and 0.95 for 5000, 500 cells and 50 cells, respectively (Fig. S2b). The method's consistency was further validated by strong insulation score correlations, ranging from 0.84 for 5000 cells to 0.79 for 50 cells (Fig. S2c).

In summary, HiChew represents a significant advancement in chromatin conformation capture technology for samples with limited input material. This innovative method demonstrates enhanced efficiency, capturing valid pairs 4–6 times more effectively than existing techniques such as Dip-C. By enhancing data yield while preserving sensitivity and accuracy, HiChew offers a cost-effective solution for researchers in the field of genomics and epigenetics.

### The high throughput single nuclei HiChew (snHiChew)

HiChew has demonstrated enhanced sensitivity in detecting chromatin conformation, even with limited cell numbers. Building on this success, we sought to extend HiChew's capabilities to single-cell analysis. Previous methods such as Dip-C, GAGE-seq, and LiMCA employ unenriched approaches, resulting in substantial data waste (84–92%, Fig. S3a, deduplicated valid pairs per read). While conventional biotin-based snHi-C achieves > 75% valid data, it sacrifices capture sensitivity and yields limited unique pairs at higher sequencing depths (Fig. S3ab, deduplicated valid pairs). Recognizing the need for a method combining both sensitivity and efficiency, we adapted HiChew for single-cell chromatin conformation capture using post-PCR enrichment. This innovation led to the development of snHiChew, a high-throughput and efficient technique optimized for single nuclei analysis.

The snHiChew method builds upon the principles of HiChew, following a series of refined steps. Initially, we perform crosslinking to preserve chromatin structure. Subsequently, we digest the DNA and ligate it into concatemers, effectively capturing the chromatin conformation. We then utilize HpyCH4V to digest the DNA concatemer, preparing it for barcode attachment. Following this, we distribute the cells across 96 wells. The process concludes with dA-tailing and ligation of the first round of barcoded adapters, as illustrated in Fig. 2a.

At this phase, we gather the nuclei and segregate them into various groups on the secondary plates. We then conduct PCR to affix the secondary barcode (Fig. 2a). Utilizing the split-and-pool method [13, 14], we theoretically have the capacity to produce 147,456 barcodes (384 × 384), potentially accommodating up to 20,000 cells. In a smaller batch test, a 96 × 96 barcode system could potentially handle around 1000 cells.

After PCR amplification of the entire genome library, we utilize the methyltransferase to mark the junction scars with m6A. Then, we employ the m6A antibody to enrich the valid pair reads (Fig. 2a). It is worth noting that we should steer clear of the GATC motif when designing the barcode. This is to avoid being targeted by the methyltransferase, which could potentially skew the data.

To evaluate snHiChew's accuracy and efficiency in single-cell identification, we conducted experiments using a mixture of HEK293T and NIH/3T3 cells. In our initial batch of 400 cells processed through snHiChew, we observed 27 doublets, yielding a collision rate of 0.059 (Fig. 2b). A subsequent batch of 1,000 cells (Table S2) showed a similar collision rate of 0.062 after doublet filtering (Fig. S3c). Using UMAP [15] clustering based on eigenvalue, we achieved clear separation between the two cell types (Fig. S3d).

To assess snHiChew's data quality, we compared it with two established methods: Dip-C (an unenriched approach) and snHi-C (a biotin enrichment technique). We used 200 HEK293T cells and 400 GM12878 cells for this evaluation (Fig. S4a, Table S3, Table S4). To ensure data reliability, we implemented stringent quality control measures, analyzing reported pairs (Fig. S4c), cis–trans ratios (Fig. S4b), distance decay curve profiles (Fig. S4d), AB compartments (Fig. 2c), and TAD correlations (Fig. S4e). All three methods showed similar cis–trans ratios and distance decay curves. Like bulk samples, pseudo-bulk snHiChew closely replicated bulk Hi-C findings across key parameters: AB compartments (correlation coefficient: 0.86–0.91) and TAD insulation (correlation coefficient: ~ 0.80) (Fig. 2c, Fig. S4f). These data also exhibited high reproducibility between replicates (Fig. 2c, Fig. S4f). At higher resolution, snHiChew and Dip-C (LiMCA) showed high consistency in signal enrichment for loops, and snHiChew demonstrated category distributions closer to the gold standard Hi-C (Fig. S3ef). These results indicate that snHiChew effectively distinguishes individual cells while capturing chromatin conformation with accuracy comparable to established bulk methods.

To evaluate data usage efficiency, we normalized all cells to approximately 100,000 reads per cell. In HEK293T cells, Dip-C and snHi-C sequencing yielded valid pair ratios of approximately 12–20% and 70 ~ 90%, respectively, while snHiChew achieved around 45% (Fig. 2d). In GM12878 cells, snHiChew reached a ~ 50% valid pair ratio, which is fourfold higher than Dip-C but 45% lower than snHi-C (Fig. 2d). As expected, snHi-C, which employs biotin purification, exhibited the highest data usage efficiency but also the highest duplication rate, indicating an abundance of non-informative duplicated reads (Fig. 2e, Fig. S4b). Our analysis further reveals that snHiChew outperforms recent technologies—LiMCA, and GAGE-seq—by 4–7 times, producing the highest recovery of informative contacts from gold standard HiC with a comparably low duplication ratio (Fig. S3a).

Next, we assessed library complexity. The presence of duplicated pairs at low sequencing depths in snHi-C indicates lower library complexity (Fig. 2e, lower panel), resulting in fewer informative contacts and more rapid saturation as sequencing depth increases. To examine this phenomenon, we analyzed the deduplicated valid pairs across increasing sequencing depths. snHi-C quickly reached its saturation point of 28,988, whereas snHiChew's saturation point of 7,293,045 significantly surpassed that of snHi-C, demonstrating enhanced capture sensitivity for preserving informative valid pairs (Fig. 2e, upper panel). However, Dip-C's saturation point remains undetermined because additional valid contacts would be needed to approach this point. Dip-C and snHiChew have comparable valid pair duplication rates across all sequencing depths, suggesting similar library complexity between these two methods. These results support our previous conclusion from bulk samples (Fig. 1c).

snHiChew significantly enhances single-cell sequencing resolution through improved data generation efficiency. Our analysis reveals that with 500,000 valid pairs per cell, snHiChew provides coverage exceeding 80% of 10 kb genomic bins and approximately 70% of 5 kb genomic bins (Fig. 2f). In comparison to other technologies at equivalent read numbers, snHiChew demonstrates improved coverage, surpassing LiMCA by 25% and snHi-C by 80% at 5 kb resolution (Fig. S3b). This enhanced resolution capability allows for the identification of a greater number of enhancer-promoter interactions in individual cells compared to existing technologies.

Notably, we achieved this high resolution while significantly reducing sequencing costs, without necessitating a substantial increase in sequencing depth. This advancement has the potential to significantly enhance our understanding of transcriptional regulation within cells. In terms of data utilization efficiency, maximum contacts captured, and resolution quality, snHiChew has demonstrated enhanced performance compared to existing methodologies. Our rigorous validations and benchmarking processes confirm the enhanced quality of data produced by snHiChew, consistently yielding high-resolution contact maps for individual cells.

### TAD melting with specific gene activation in B compartments

Following the development of our high-resolution snHiChew technique, we aimed to utilize this advanced technology to explore complex chromatin phenomena. The genome is primarily organized into two major compartments: A and B [16]. The B compartment, typically associated with heterochromatin, is proximal to the nuclear lamina and generally linked to transcriptional repression. However, previous research has shown that genes within the B compartment can still exhibit expression [17]. The intricate relationship between chromatin organization and gene expression in this context remains a crucial area of investigation in 3D genomics.

**Fig. 3:**
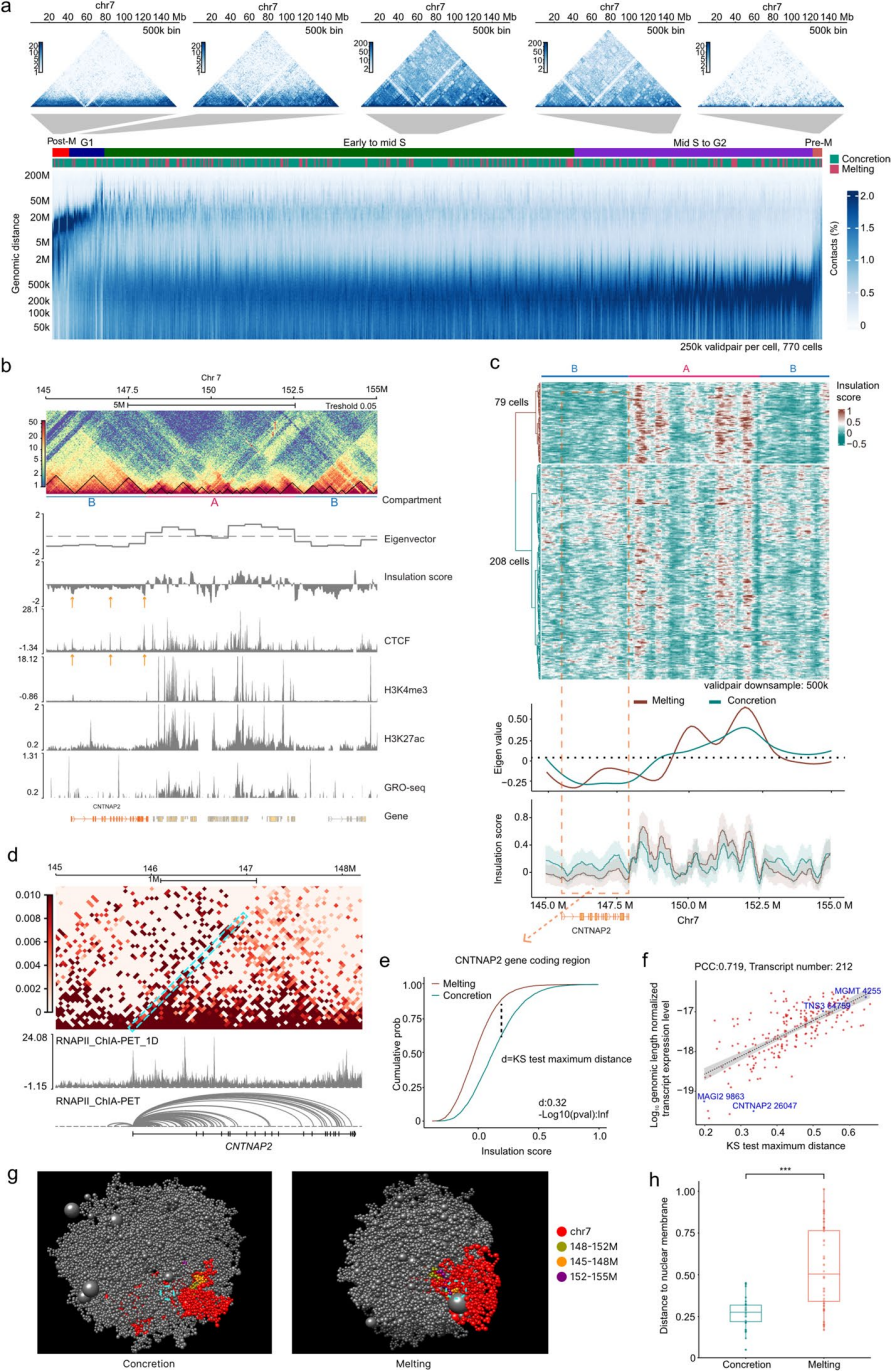
Analysis of chromatin organization during tanscriptional activity. **a** Single-cell contact decay profiles ordered by Repli-seq peaks labeled early to late cell-cycle phasing, with melting state and approximate cell-cycle phases shown on top. Each column represents a single cell. **b** Genomic track view of chromatin compartmentalization within chr7:145 M-155 M, incorporating Hi-C contact matrices alongside corresponding eigenvector values, insulation metrics, *CTCF* binding profiles, histone modification patterns (H3K4me3, H3K27ac), transcriptional activity, and gene structural annotations. **c** Single cell insulation score patterns across distinct chromatin states, illustrating cellular organization in melting (red) and concretion (teal) configurations, with smoothed line graph showing insulation score distribution and compartment transitions. The error bar represents the standard deviation (SD). **d** Correlation map displaying chromatin interactions within the chromosomal region chr7:145 M-148 M, with color intensity representing interaction frequency (darker red indicating stronger interactions). Below the map is a track showing RNA polymerase II ChIA-PET data (RNAPII_ChIA-PET_1D and RNAPII_ChIA-PET), illustrating transcriptional activity and polymerase-based interactions across this region. At the bottom is a genomic annotation of the CNTNAP2 gene structure. **e** Cumulative probability distributions of single-cell insulation scores at the genomic region of gene CNTNAP2 across the melting and concretion state cells. A one-sided Kolmogorov–Smirnov (KS) test was performed to obtain the maximum distance between the two distributions. **f** Correlation analysis of relationships between KS test max distance of single cell insulation score and genomic span normalized transcript RSEM expected count (PCC: Pearson correlation coefficient). Specific gene examples, including CNTNAP2, as well as MGMT, TNS3, and MAGI2 which represent different expression levels, are labeled to illustrate the distribution across the correlation. **g** Three-dimensional structural visualization of chromatin architecture across specified regions (chr7: 145 M-148 M, 148–152 M, and 152 M-155 M). **h** Structural analysis of smallest Euclidean distance (calculated by XYZ coordinates) of cLAD to 3D modeling constructed TAD beads in chr7: 145 M-148 M region of chrom3D models, comparing concretion (cell number = 3) and melting (cell number = 3) states, revealing statistically significant variations in nuclear membrane proximity. Two-sided Wilcoxon signed-rank test was used, *** indicates *P* < 0.001

To investigate this phenomenon, we conducted a comprehensive study using 1200 HEK293T cells. After removing doublets, we obtained approximately 1178 valid cells (Fig. S6a), yielding between 250,000 and 2 million unique valid pairs per cell (valid pair ratio: 0.44, reported pair ratio: 0.66, Table S5). We implemented stringent quality control measures, including cis–trans ratio analysis (Fig. S6b, Table S6), false positive rate (Fig. S6c), distance decay curve evaluation (Fig. S6d), and comparisons of AB compartments, TAD insulation, and loop structures (Fig. S6e-g). The pseudo-bulk analysis of these cells demonstrated robust correlation with gold standard Hi-C data, yielding an eigenvalue correlation of 0.88 and insulation score correlation of 0.86 (Fig. S6e,f). Furthermore, we calculated the early replication ratio based on established methods [18], ranking cells from early to late replication stages. We observed a smooth, cyclical transition in the distribution of genomic contact distances from short to long range, similar to previously observed cell cycle phenomena in mouse embryonic stem cells [18] (Fig. 3a). These results substantiate the high quality and reliability of our single-cell data.

Our study identified some long coding genes situated within the B compartment. We chose a typical region, spanning from 145 to 155 Mb, as a representative example (Fig. 3b). The stretch from 145 to 148 MB, encompassing the long gene *CNTNAP2* with a genomic size of 2.3 MB, was located within the B compartment and covered by 3 TADs. The subsequent region, extending from 148 MB to 152.5 MB, represented another A compartment containing over 30 genes. The final section, from 152.5 MB to 155 MB, was again within the B compartment. The insulation score showed three drops in the segment from 145 to 148 MB, with the presence of *CTCF* peaks on the TAD boundary further confirming the existence of the TADs (orange arrow). Interestingly, we observed some active histone modifications, such as H3K4me3 signal on the *CNTNAP2* promoter and H3K27ac on the gene body. Furthermore, we observed the transient RNA expression of *CNTNAP2* (GRO-seq). While *CTCF* binding and TAD structures are known to be dynamic, the efficient transcription of such a long gene through multiple TADs raises interesting questions about the coordination between transcriptional machinery and chromatin organization.

We hypothesized that gene transcription may facilitate DNA relaxation and TAD dissolution. To examine this possibility, we standardized our analysis by downsampling valid pairs to 500 k per cell, reducing potential depth-related variability. In HEK293T cells, the chromatin contact data supported separation into two clusters based on insulation scores (Fig. 3c). The first cluster exhibited decreased insulation scores in the B compartment region (red dashed box) with correspondingly elevated scores in the adjacent A compartment, indicating a dissolution state (melting label—reduced insulation and merging with neighboring TADs) within the 145 Mb–148 Mb B compartment region. In contrast, the second cluster demonstrated the inverse pattern in the B compartment, suggesting a solidification state (concretion label—increased insulation and forming more small TADs) in this genomic region. This clustering did not appear to be driven by major differences in sequencing bias or library quality, as both clusters demonstrated similar cis–trans ratios and valid pair ratios (Fig. S7f). We also did not observe a strong dependence on cell cycle progression, as the clusters showed comparable replication scores and distributed across G1-G2 phases (Fig. 3a and Fig. S7eg). It is important to note that this differentiation becomes less discernible when valid contacts per cell decrease below 250,000 (Fig. S7a).

RNA polymerase II ChIA-PET analysis provides additional evidence supporting the dynamic dissolution of TADs during transcription. Our examination revealed RNA polymerase II-mediated interactions between the transcription start site and various locations throughout the gene body, with 31% traversing TAD boundaries and increasing intra-TAD contacts (Fig. 3d bottom). Furthermore, we observed the absence of TAD boundary formation in the ChIA-PET data (Fig. 3d top), indicating that TAD structures are not maintained during active transcription. These observations are consistent with the possibility that active transcription is associated with reduced TAD boundary insulation along the transcriptional pathway.

Our hypothesis aligns with existing research on the relationship between chromatin structure and gene expression. Previous studies across various cell types have demonstrated a correlation between TAD melting and gene activation [19]. This suggests that the melting phenomenon may be a general occurrence. To establish the relationship between melting status and RNA expression, we investigated a broader range of genes to determine if expression levels are positively associated with the degree of melting.

Our analysis identified 751 genes showing evidence of variable melting/concretion-like states across cells (Fig. S7b, 8def examples). We identified that 746 gene bodies contain at least one TAD boundary (Fig. S7d). Additionally, we detected RNA polymerase II-mediated interactions between transcription start sites (TSS) and transcription end sites (TES), indicating that active transcription processes traverse TAD boundaries (Fig. S7c). To assess the relationship between chromatin structure melting and gene expression, we employed the methodology established by Winick-Ng et al. (2021). We quantified the maximum distance between cumulative curves for each transcript's genomic region (Fig. 3e) and implemented the Kolmogorov–Smirnov (KS) test to determine the degree of melting states, concentrating on transcripts with expression levels (RSEM expected count > 2). We observed a positive correlation (PCC = 0.773) between normalized expression levels (by genomic region length) and KS test distance, consistent with an association between larger melting-like changes and higher gene expression levels (Fig. 3f). These results align with previous research and further elucidate the sophisticated relationship between chromatin structure dynamics and transcriptional activity.

We then investigated the relationship between chromatin structure melting and the movement of chromatin toward the active center. Eigenvalue analysis of each cluster indicated a modest increase in the melting status compared to the concretion status within the 145–148 MB B compartment (Fig. 3c). This observation may reflect modest repositioning of the gene away from the nuclear lamina, a region typically associated with transcriptional repression [20]. Three-dimensional modeling of individual cells in melting and concretion states revealed that cells in the melting status generally maintain a distance from the nuclear membrane approximately twice that of cells in the concretion status (Fig. 3gh). The intron FISH experiments are consistent with transcription occurring at locations that are less proximal to the nuclear membrane (Fig. S11). This finding implies that actively expressed *CNTNAP2* is likely situated further from the LAD (lamina-associated domains).

To further examine the relationship between RNA expression and chromatin conformation changes in another cell line, we analyzed LiMCA data from GM12878 cells. Our analysis revealed that cells exhibiting melting status demonstrated substantially higher gene expression levels compared to those in concretion status (Fig. S8abc). However, it is important to note that chromatin conformation changes are primarily associated with transient transcriptional events, while cellular RNA levels are subject to multiple regulatory mechanisms. Consequently, the observed changes in chromatin structure did not consistently correlate with significant variations in overall RNA levels, as initially hypothesized.

This section examines the relationship between chromatin structure and gene activation in the B compartment. We observe that reduced TAD insulation (melting-like behavior) is associated with higher gene expression and chromatin accessibility in this dataset. We also observe differences in inferred positioning between melting- and concretion-like states. Together, these results suggest a relationship between chromatin organization, accessibility, and gene activity that may refine models of transcription within heterochromatin-associated compartments. Advanced techniques like snHiChew provide insights into these subtle yet significant changes in chromatin organization and their potential impact on gene regulation.

### Effects of *CTCF* knockdown and transcription on gene expression and chromatin structure

To further explore the interplay between chromatin structure and gene expression, we employed targeted interventions to disrupt the concretion process and transcription-related melting. Our previous findings, corroborated by studies from Rao [21] and Tang [22], suggest that CTCF is predominantly situated between TADs in the B compartment. This compelling observation led us to investigate whether a decrease in CTCF protein expression might enhance the frequency of chromatin melting.

**Fig. 4:**
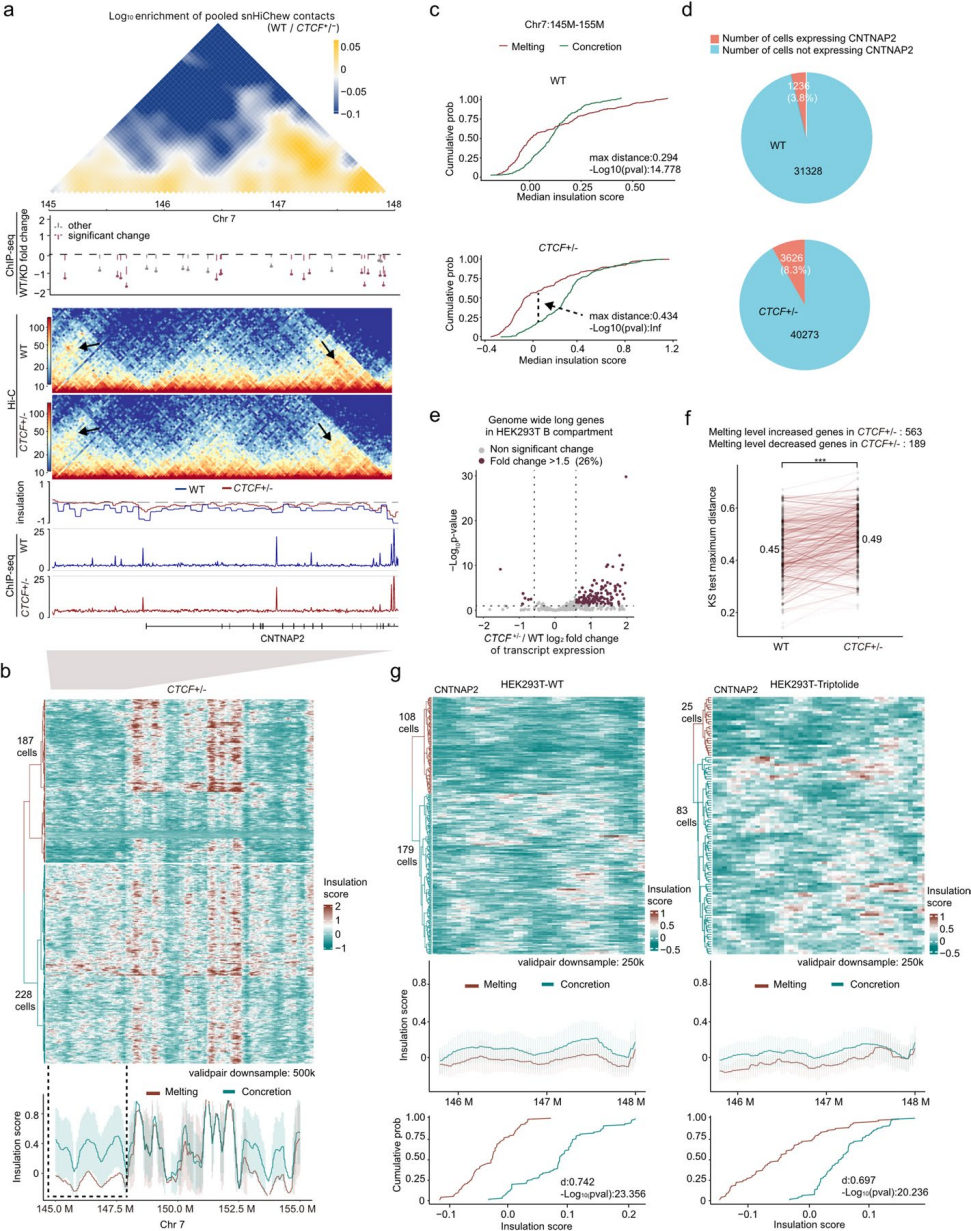
*CTCF* knockdown and transcriptional impact on chromatin organization. **a** Genomic track view of chromatin structure in chr7: 145–148 M region, featuring comparative contact matrices of HEK293T wild-type and *CTCF* ± cells, alongside CTCF ChIP-seq differential analysis, contact matrices, insulation scores, and *CTCF* binding patterns. **b** Heatmap of 415 HEK293T *CTCF* ± cells insulation score grouped by hierarchical clustering and visualized on a red-cyan spectrum. Mean insulation scores for chr7: 145 M-155 M are presented in the curve below. The error bar represents the standard deviation (SD). The median insulation scores of each genomic bin in melting or concretion cell groups are used. **c** Cumulative probability distributions of single-cell insulation scores at the genomic region of chr7:145 M-155 M across the melting and concretion state cells of wild-type (top) and *CTCF* ± (bottom) samples, yielding KS test values of d = 0.294 and d = 0.434 respectively, indicating enhanced chromatin melting in HEK293T *CTCF* ± cells. **d** Distribution analysis of CNTNAP2 expression across HEK293T wild-type and *CTCF* ± populations with corresponding cell counts from single-cell RNA-seq datasets. **e** Volcano plot showing bulk RNA-seq differential expression of genome-wide long genes in HEK293T B compartment between HEK293T *CTCF* ± and wild-type samples, displaying log10 fold changes relative to statistical significance (*p* < 0.05). **f** Pairwise evaluation of melting level (KS test maximum distance) differences between wild-type and *CTCF*-deficient cells (contacts > 250 K), showing a significant increase in insulation score curve separation for the genome-wide long genes in HEK293T B compartment. Same gene of the two samples is connected with a line. A two-sided Wilcoxon signed-rank test was used (*** *p* < 0.001). **g** Heatmap illustrating the insulation scores of HEK293T single cells treated with DMSO and triptolide at the CNTNAP2 genomic region. The data is grouped by hierarchical clustering and visualized on a red-cyan spectrum, indicating a reduced chromatin melting state under transcriptional inhibition. Below the heatmap, a line graph displays the mean insulation scores, alongside cumulative probability distributions of single-cell median insulation scores for each genomic bin in both melting and concretion cell groups

To investigate the effects of reduced *CTCF* expression, we utilized *CTCF* ± cell lines with significantly lower CTCF protein and RNA levels (Fig. S9a-e). ChIP-seq analysis identified approximately 23,000 peaks with decreased *CTCF* binding (Fig. S9f,g, Table S7), while RNA-seq data showed upregulation of 571 genes (Fig. S9h), including a 1.5-fold increase in *CNTNAP2* expression. We performed snHiChew sequencing on 1,015 *CTCF* ± cells, obtaining an average of 50,000 valid pairs per cell (Fig. S6a,b, Table S8). Insulation analysis revealed a modest decrease in the insulation score at the TAD level in *CTCF* ± cells (Fig. 4a), correlating with reduced *CTCF* binding peaks in TAD boundary regions. Contact map analysis demonstrated that *CTCF* ± cells exhibited a higher prevalence in broader TAD regions (Fig. 4a), whereas wild-type cells showed more pronounced clustering in small TADs. This observation suggests an increased frequency of chromatin melting in *CTCF* ± cells, underscoring the relationship between reduced *CTCF* binding and alterations in chromatin architecture.

Our single-cell clustering analysis revealed that the proportion of cells in a melting state increased significantly from 27 to 45% (Figs. 3c, 4b). KS analysis demonstrated a substantial shift in the single-cell insulation score cumulative curve (from 0.29 to 0.43) within the chromatin structure of genomic region Chr7:145 M–155 M following *CTCF* downregulation (Fig. 4c). This structural alteration corresponded directly with changes in gene expression patterns. Analysis of single-cell RNA-seq data [23] indicated a twofold increase in *CNTNAP2* expression frequency in *CTCF* ± cells compared to wild-type (Fig. 4d). Furthermore, genome-wide examination identified 563 of 752 HEK293T B compartment long genes that displayed enhanced melting patterns in the *CTCF* ± cell line, with 504 of these genes showing elevated transcript expression levels (195 genes with significant expression changes, Fig. 4e,f, examples Fig. S9i). Genes with higher expression fold changes also showed significantly greater KS distances than genes with lower changes (Fig. S9l). These results establish a robust correlation between reduced CTCF-mediated chromatin insulation and increased gene expression activity.

To investigate the relationship between transcription and chromatin structure dynamics, we treated cell cultures with triptolide for 2 h before snHiChew analysis. The treatment effectively downregulated 17,608 genes (Fig. S9j). Melting status decreased substantially from 39% in wild-type cells to 23% in treated cells (Fig. 4g, Table S9). Cumulative plot analysis showed that transcription inhibition caused the melting curve to align more closely with the concretion state, indicating decreased chromatin melting (Fig. 4g). This led to increased concretion status and elevated insulation scores—our analysis demonstrated a threefold increase(Fig. S9k). These results establish an association between transcription inhibition and chromatin structure dynamics, while also revealing subtle regional variance that suggests sophisticated regulatory mechanisms requiring further exploration.

Our study revealed that *CTCF* down-regulation increases chromatin melting and gene expression, while transcription inhibition reduces the melting state. These findings highlight the complex relationship between chromatin structure, transcription, and gene regulation, advancing our understanding of chromatin dynamics and their role in cellular processes.

### Applying snHiChew for testis samples

Following our successful implementation of snHiChew in cultured cells, we extended our investigation to examine its performance in more complex tissue environments, specifically in spermiogenesis analysis. This investigation marks the first application of single-cell chromatin conformation analysis in spermiogenesis research. Utilizing our established spermiogenesis purification protocols [23, 24], we performed snHiChew analysis on a cohort of ~ 1,100 spermiogenesis cells, yielding an average of 275 K reads per cell (Table S10).

**Fig. 5:**
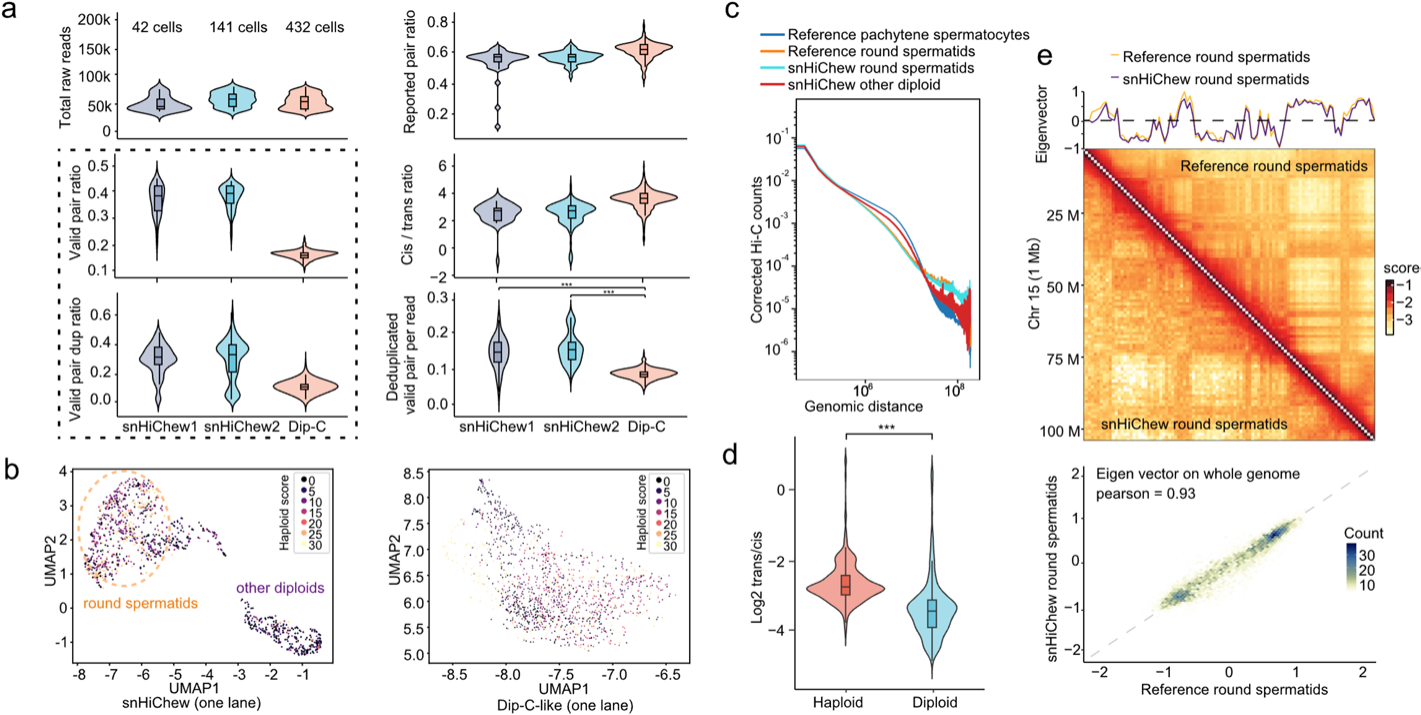
Implementation of snHiChew in testis sample. **a** Single cell metrics across experimental conditions in testis samples, utilizing violin plots to illustrate the distribution of critical quality parameters: total raw reads processed, valid pair ratio, valid pair duplication ratio (left), reported pair ratio, log2 cis–trans ratio, and unique valid pair/read ratios (right) across experimental cohorts. Number of cells for each sample is indicated in the first graph. Two-sided Wilcoxon signed-rank test was used, *** indicates *P* < 0.001. **b** UMAP-based visualization representing snHiChew contacts from approximately 1,100 testis cells, with clusters labeled by haploid scoring metrics to distinguish spermatocyte/spermogonia (*n* = 297) from round spermatid populations (*n* = 877). Dip-C control is on the right side. **c** Contact probability distance decay curve comparing reference pachytene spermatocytes (blue), reference round spermatids (orange), snHiChew round spermatids (green), and additional diploid cells (red). **d** Violin plots illustrating the distribution of log-transformed trans/cis ratios between round spermatids (haploid, *n* = 877) and spermatocyte/spermogonia (diploid, *n* = 297) populations. Two-sided Wilcoxon signed-rank test was used, *** indicates *P* < 0.001. **e** Comparison of chromatin architectural patterns between bulk Hi-C and pooled snHiChew datasets for round spermatids. Upper section: Contact matrices illustrating chromosome-wide interactions at 500 kb resolution with the corresponding eigenvector track at top. Lower section: Correlation analysis of eigenvector values between snHiChew data and established bulk Hi-C profile (Pearson correlation coefficient: 0.93)

Quality control analysis demonstrated that tissue samples achieved a valid pair ratio of approximately 40%. While this was lower than that of our cultured cells, it significantly outperformed the parallel Dip-C experiment by a factor of three, validating snHiChew's effectiveness for tissue sample analysis (Fig. 5a, Fig. S10b,d QC). Concurrent analysis revealed elevated duplication rates at 50 k reads sequencing depths in tissue samples (Fig. 5a), indicating reduced valid contact information per cell compared to cultured cells. This reduction in efficiency was expected given the unique characteristics of spermiogenesis samples, particularly round spermatids with their compact nuclear structure. The dense nuclear organization might present a physical barrier to enzyme penetration, thereby limiting digestion efficiency. Gel electrophoresis analysis of the in vivo digested genome confirmed lower digestion efficiency compared to cultured cells (Fig. S10a).

Using UMAP analysis, we identified two distinct cell clusters (Fig. 5b). To validate our classification of spermatocyte/spermatogonia and round spermatids, we conducted a quantitative assessment of sex chromosome content per cell. Given that pachytene spermatocytes possess diploid or tetraploid chromosomes, they naturally demonstrate a lower X/Y chromosome percentage relative to haploid round spermatids (detailed methodology in methods section)(Fig. S10c). Through this analytical approach, we successfully distinguished the two clusters: spermatocyte/spermatogonia and round spermatids, with higher haploid scores correlating with increased probability of spermatid identification.

While the haploid score serves as an informative metric, sequencing variations in sex chromosome read counts create some overlap between diploid and haploid cell classifications (Fig. S10c), resulting in heterogeneous UMAP visualization patterns. However, the haploid score remains a reliable indicator for round spermatid identification. To substantiate our classification, we performed aggregated pseudo-bulk analysis comparing round spermatids with spermatocytes/spermatogonia. The results demonstrated that round spermatids possess a markedly elevated trans/cis ratio relative to spermatocytes/spermatogonia (Fig. 5d). Furthermore, distance decay analysis revealed that spermatids exhibit characteristically shorter-distance interactions compared to spermatocytes (Fig. 5c, Fig. S10d), aligning with previously documented findings [25]. Contact map analysis provided additional confirmation, revealing predominantly short-distance interactions in round spermatids that correspond to nuclear condensation patterns (Fig. 5e). The robust correlation between aggregated spermatid eigenvalues and established literature findings (PCC: 0.93) provides further validation of snHiChew's clustering precision. In further analysis of AB compartments in spermiogenesis, we found that most regions invert from B compartment to A compartment. This occurs because pachytene spermatocytes remain in the meiosis stage, where the Hi-C structure is abnormal (Fig. S10ef). After progressing to round spermatids, the Hi-C structure is reestablished [26]. GO term analysis showed that the inverted compartments were primarily enriched for testis-related terms.

In conclusion, snHiChew was successfully applied to spermiogenesis cells, enabling effective classification of cell types and providing valuable insights into chromatin organization during this critical developmental process.

## Discussion

In this study, we introduced highly efficient technologies, HiChew and snHiChew, which significantly address a major issue in this field: the inefficient incorporation of biotin enrichment into single cell analysis, resulting in significant loss of valid contacts. Traditionally, many researchers have resorted to Dip-C that rather sequences the whole genome without enrichment, leading to a waste of approximately 90% of the data. This approach, while enabling high sensitivity to capture more interactions, has dominated the field for many years.

Previously, the idea of enriching the ligated fragments after PCR, which could generate valid contacts without sacrificing DNA loss, was not widely explored. Our method, HiChew, solves this long-standing problem and is of increasing importance in current studies.

The valid pair ratio, calculated by dividing valid pairs by reported pairs, serves as a more reliable metric for comparing methodological efficiency across different technologies. This parameter remains stable across sequencing depths and effectively represents method efficiency. While cell type variations in contact-to-genome ratios can influence this parameter, our observations across HEK, GM12878, and testis cells show variations typically remain within 10%, excluding extreme cases.

To further validate efficiency measurements, we analyzed the relationship between deduplicated valid pairs and sequencing reads through saturation curves. These curves provide valuable insights into both the ratio of unique valid pairs per read and overall library complexity. However, generating these parameters requires substantial resources, and saturation points vary significantly by cell type. For instance, testis samples demonstrated 2 × higher duplication rates (Fig. 5a, Fig. S4b) and lower maximum contacts compared to HEK293 or GM12878 cells, a common challenge in single-cell 3 C experiments [27, 28]. This limitation likely stems from reduced enzyme penetration through the nuclear membrane in tissue cells, highlighting the need for optimized protocols across different tissue types.

The effectiveness of our enrichment method is directly correlated with digestion efficiency, as undigested GATC motifs constitute significant byproducts during immunoprecipitation. Our systematic evaluation demonstrated that overnight digestion protocols significantly improved enrichment outcomes. We conducted experimental trials using Tn5 for adapter attachment during the in vivo snHiChew process, achieving a notable 70% valid pair ratio (data not published). However, the in vitro extracted DNA Tn5 attachment method did not demonstrate comparable advantages. Furthermore, this approach was unable to accurately reproduce the contact map patterns observed in standard Hi-C, an outcome likely attributable to Tn5's selective binding to accessible regions within the ligated concatemer, as documented in recent publications [29]. In light of these methodological constraints, we elected to exclude Tn5 from our standard snHiChew protocol. Nevertheless, Tn5-based methodologies may offer particular utility in investigations focusing on open chromatin interactions.

We conducted size selection experiments to analyze immunoprecipitation preference of varying fragment lengths (Fig. S5g). Our analysis revealed that 500 bp libraries demonstrated a 10% higher efficiency compared to 300 bp libraries. However, subsequent comparisons of pre- and post-immunoprecipitation sizes showed only a minimal 25 bp shift. Gel electrophoresis analysis further confirmed the absence of significant size alterations before and after immunoprecipitation. Based on these observations, we concluded that the variation in valid pair ratios is not attributable to antibody preference for fragment size. Rather, this difference can be explained by two technical factors: shorter fragments have a reduced probability of containing ligation scars, and fragments below 50 bp present challenges for accurate genome alignment.

Our methodology does have some limitations. Specifically, we're confined to choosing the restriction enzyme that pairs with the methyltransferase for marking the ligation scar, such as DpnII-dam methyltransferase and AluI-AluI methyltransferase. In our research, we employed DpnII and AluI to evaluate the abilities of HiChew. It's important to recognize that the commercial availability of these enzymes is on the rise, with roughly 20 different enzymes currently on the market. This could broaden the application of our methodology to a range of conditions. Another aspect to ponder is whether the resolution of HiChew can be improved by utilizing multiple enzyme digestion. The theoretical resolution of the DpnII digestion is approximately ~ 700 bp. If our goal is to break down genome into smaller fragments for better resolution, we might think about using a blend of multiple enzymes, such as a combination of DpnII and AluI. This could theoretically enhance the resolution to ~ 400 bp, albeit at the cost of some sensitivity.

Topologically Associating Domains (TADs) exhibit dynamic structural properties that are dependent on *CTCF* binding, as demonstrated in previous research [30]. Our *CTCF* knockdown experiments revealed a correlation between chromatin structural changes, gene expression patterns, and *CTCF* binding activity. In the B compartment, we observed that decreased TAD insulation and reduced *CTCF* binding correlated with enhanced gene expression, though the causality remains unclear. However, our current analysis lacks definitive evidence establishing a direct link between transient gene expression and chromatin conformation changes at the single-cell level, as these technologies are still in development. We aim to address this limitation in future studies. While our findings suggest interesting associations, a comprehensive investigation of these molecular mechanisms extends beyond the scope of this methodological study.

Our laboratory has successfully demonstrated snHiChew's robust performance in animal tissue analysis, particularly in testis samples. The versatility of HiChew technology extends to its integration with established methodologies such as HiChIP [31], traditionally known for their substantial cell requirements. Through HiChew implementation, HiChIP protocols have been optimized to require only a single immunoprecipitation step, thereby enhancing efficiency and reducing cell input requirements while maintaining high success rates. This optimization significantly advances the accessibility of these sophisticated methodologies. The technology's adaptability further extends to integration with various multi-omics platforms, including HIRES (RNA + Hi-C) and Methyl-HiC (methylation + Hi-C). A recent advancement in our laboratory has been the successful incorporation of this technology into the 10X system, resulting in streamlined protocols and enhanced data quality [32]. These developments collectively establish HiChew as a sophisticated and efficient methodology for chromatin conformation analysis.

## Conclusions

We present HiChew and snHiChew, novel chromatin conformation capture methods that achieve ~ 50% valid pair ratios—a sixfold improvement over traditional unenriched approaches. The single-cell variant, snHiChew, maintains 45–50% efficiency while enabling high-resolution mapping at 5–10 kb with 70–80% bin coverage. By combining efficient sticky-end ligation with post-PCR methylation-based enrichment, HiChew addresses the longstanding inefficiency of biotin enrichment in single-cell analysis, transforming previous 90% data waste into a highly efficient methodology. The technology demonstrates robust performance across diverse cell types and tissues, including successful application to complex spermiogenesis samples. HiChew's adaptability extends to integration with established platforms such as HiChIP, HIRES, Methyl-HiC, and the 10X system, significantly reducing cell input requirements while maintaining high data quality. These advances position HiChew as a transformative tool for investigating 3D genome organization at single-cell resolution.

## Methods

### Cell type and cell culture

NIH/3T3 cells (RM02840, Abclonal, Wuhan, China), HEK293T WT cells and *CTCF* Knockdown 293 T cells (RM01849, Abclonal, Wuhan, China) were cultured in DMEM (Gibco, Cat. No. 11965092) medium supplemented with 10% FBS (Gibco, Cat. No. 10099141) and 1% penicillin–streptomycin (Gibco Cat. No. 15140122) under the conditions of 37 °C, 5% CO$_2$. GM12878 cells (GM12878, Coriell Institute; mycoplasma tested) were cultured in RPMI1640 (Gibco, Cat. No. C22400500BT) medium supplemented with 15% FBS and 1% penicillin–streptomycin under the conditions of 37 °C, 5% CO$_2$. For transcription inhibition experiment, HEK293T cells were treated with 1 μM triptolide for 2 h. Mycoplasma test was routinely performed using detection kit (MycAway).

### Purification of spermatogenic cells

All mice utilized in this study were of the C57BL/6 J background and were maintained under specific pathogen-free conditions within a temperature- and humidity-controlled animal facility at Jinan University. The animal operation protocol for this study received approval from the Laboratory Animal Welfare and Ethics Committee of Jinan University. Purification of spermatogenic cells was performed as previously description [24]. Briefly, after collection and decapsulation, two testes were digested in EKRB buffer containing collagenase to disperse the testicular cells. The dispersed cells were washed, digested with trypsin and DNase I, and washed three times. Cells were resuspended in 1 × PBS solution and processed to fixation steps.

### HiChew experimental workflow

#### Cell fixation

Culture medium was removed, and cells were washed once with 1 × PBS. Trypsinization was carried out by adding 2 mL of 0.025% trypsin to a 10-cm culture dish, and incubated at 37 °C until detachment. Detached cells were collected in 4 mL of complete DMEM medium and centrifuged at 400 × g for 3 min. The cell pellet was washed twice with 1 × PBS, and fixed with 2 mL of 1% formaldehyde (16% wt/vol, ThermoFisher, Cat. No. 28908) in PBS at room temperature for 10 min. Fixation was terminated with 100 μL of 2.5 M glycine for 5 min. Cells were centrifuged at 800 × g for 5 min, washed twice with 1 × PBS, and resuspended in 2 mL of PBS containing 3 mM ethylene glycol bis(succinimidyl succinate) (EGS, Thermo, Cat. No. 21565) for a second fixation at room temperature for 45 min. Fixation was terminated by adding 400 μL of 2.5 M glycine to quench the reaction, and the tube was incubated for 5 min at room temperature, followed by centrifugation and washing twice with 1 × PBS. Fixed cells were either processed immediately or flash-frozen in liquid nitrogen for storage.

#### Cell lysis

Cells were lysed in 500 μL of permeabilization buffer (10 mM Tris–HCl [pH 8.0], 10 mM NaCl, 0.2% CA-630, 1 × protease inhibitors cocktail) per 2 × 10$^6$ cells and incubated on ice for 10 min. After centrifugation at 800 × g at 4 °C for 3 min, the supernatant was discarded, and the pellet was washed once with 1.2 × NEBuffer r3.1. The pellet was resuspended in 199 μL of 1.2 × NEBuffer r3.1, supplemented with 1 μL of 20% SDS (final concentration 0.1%), and incubated at 65 °C for 10 min. After cooling on ice for 2 min, 10 μL of 20% Triton X-100 was added to neutralize SDS, followed by incubation at 37 °C for 15 min.

#### Enzyme digestion and ligation

For restriction digestion, 200 U of DpnII (NEB, Cat. No. R0543L) or AluI (NEB, Cat. No. R0137L) was added to the reaction and incubated at 37 °C overnight. Aliquots were de-crosslinked with proteinase K at 65 °C for 1 h and analyzed by 1% agarose gel electrophoresis to confirm digestion efficiency. Digested samples were washed twice with washing buffer (1 × PBS, 1 mM EDTA, 1 mM EGTA, 0.1% Triton X-100) and resuspended in 195 μL of 1 × T4 DNA ligase buffer containing 0.1% BSA. Ligation was carried out by adding 1 μL of T4 DNA ligase (2000U/μL) (ABclonal, Cat. No. RK21500) to a final concentration of 10 U/μL and incubating at 16 °C overnight. Aliquots were de-crosslinked with proteinase K at 65 °C for 1 h and analyzed by 1% agarose gel electrophoresis to confirm ligation efficiency.

#### DNA purification, adapter attachment and PCR amplification

Nuclei were washed twice with washing buffer, resuspended in 200 μL of the same buffer, and filtered through 20-μm filter. Cell samples containing 5000, 500, or 50 nuclei were treated with proteinase K (Qiagen, Cat. No. 19133) to reverse crosslinking. The reaction was incubated at 65 °C overnight. After crosslinking reversal, DNA was purified using 1 × AMPure XP beads (Beckman Coulter, Cat. No. A63881) according to the manufacturer’s protocol. Purified DNA was processed for library preparation using the MGIEasy Universal DNA Library Prep Kit (MGI, Cat. No. 1000006985). The workflow included DNA fragmentation, adapter ligation, and PCR amplification. The adapters were replaced by Y-adapter annealed by two custom synthesis primers (5’- CGA CCA CCG AGA TGT ACA C—6 bp index—TCG TCG GCA GCG TCA GAT GTG TAT AAG AGA CAG T—3’ and 5’—/phos/CTG TCT CTT ATA CAC ATC TCC GAG CCC ACG AGA C—3’) (Table S11, Table S12) and PCR primers were replaced by HiChew-P5 primer (5′-/phos/AAT GAT ACG GCG ACC ACC GAG ATG TAC AC-3′) and i7 PCR primer (5′-CAA GCA GAA GAC GGC ATA CGA GAT—8 bp index—GTC TCG TGG GCT CGG-3′) (Table S11, Table S13). Part of the PCR product was processed to library circularization ****and sequencing to produce Dip-C like data.

#### Methylation marking

Purified DNA (1 μg) was methylated at GATC sites by incubating with 5 μL of 10 × dam Methyltransferase Reaction Buffer, 0.25 μL of 32 mM S-adenosylmethionine (SAM), and 1 μL of dam Methyltransferase (NEB, Cat. No. M0222L) in a total reaction volume of 50 μL. The reaction was carried out at 37 °C for 1 h, followed by purification with 1 × AMPure XP beads (Beckman Coulter, Cat. No. A63882).

#### Antibody preparation for IP

Ten microlitre of Protein A/G Magnetic Beads (Thermo, Cat. No. 88802) per sample were washed twice with PBST (1 × PBS, 0.1% Tween 20) and blocked with 50 μL of SuperBlock Blocking Buffer (Thermo, Cat. No. 37580) for 15 min at room temperature. Beads were washed twice with 1 × IP buffer (10 mM Tris–HCl [pH 7.5], 150 mM NaCl, 0.5% CA-630, 1 mM EDTA) and resuspended in 48 μL of 1 × IP buffer. To coat the beads, 2 μL of antibody was added (for DpnII digestion, use Anti-N6-methyladenosine (m6A) antibody (Merck, Cat. No. ABE572-I-100UG), for AluI digestion, use Anti-5-methylcytosine (5-mC) antibody (abcam, Cat. No. ab214727)), and the mixture was incubated overnight at 4 °C with rotation.

#### Immunoprecipitation

For IP, 500 ng of methylated DNA containing 20 μL of 10 μM blicking primer mix (P5 primer (5′-AAT GAT ACG GCG ACC ACC GAG ATG TAC AC-3′), P7 primer (5′-CAA GCA GAA GAC GGC ATA CGA G-3′), R1N primer (5′-TCG TCG GCA GCG TCA GAT GTG TAT AAG AGA CAG-3′), R2N primer(5′-GTC TCG TGG GCT CGG AGA TGT GTA TAA GAG ACA G-3′)) was filled to 40 μL with water and denatured at 95 °C for 5 min, chilled on ice for 2 min, and then mixed with 10 μL of 5 × IP buffer (50 mM Tris–HCl [pH 7.5], 750 mM NaCl, 2.5% CA-630, 5 mM EDTA) and 50 μL of antibody-coated beads. The mixture was incubated at 4 °C with rotation for 2 h. Beads were washed sequentially: once with medium-stringency RIPA buffer (10 mM Tris–HCl [pH 8.0], 300 mM NaCl, 1 mM EDTA, 0.5 mM EGTA, 1% Triton X-100, 0.2% SDS, 0.1% sodium deoxycholate), twice with high-stringency RIPA buffer (10 mM Tris–HCl [pH 8.0], 350 mM NaCl, 1 mM EDTA, 0.5 mM EGTA, 1% Triton X-100, 0.23% SDS, 0.1% sodium deoxycholate), and twice with 1 × IP buffer. DNA was eluted by incubating beads with 22 μL of EB buffer containing 0.05 U/mL Qiagen protease at 50 °C for 30 min. The supernatant was collected and incubated at 70 °C for 15 min to inactivate the protease.

#### Post-IP PCR amplification

For PCR amplification, 30 μL of a reaction mixture containing 25 μL of 2 × KAPA HiFi readymix (KAPA Biosystems), 1.5 μL of HiChew-P5 primer (5′-/phos/AAT GAT ACG GCG ACC ACC GAG ATG TAC AC-3′) and 1.5 μL of P7 primer (5′-CAA GCA GAA GAC GGC ATA CGA G-3′), and 2 μL of nuclease-free water was added to the eluted DNA. Amplification was performed for 6–7 cycles using a thermocycler, followed by purification with 1 × AMPure XP beads.

### snHiChew experimental workflow

The detailed protocol could be found in https://www.protocols.io/view/snhichew-x54v9rwm4v3e/v1.

#### Cell fixation, cell lysis, enzyme digestion and ligation

For HEK293T, HEK293T-*CTCF* ± and NIH/3T3 cells, ****culture medium was removed, and cells were washed once with 1 × PBS. Trypsinization was carried out by adding 2 mL of 0.025% trypsin to a 10-cm culture dish, and incubated at 37 °C until detachment. Detached cells were mixed with 4 mL of complete DMEM medium and centrifuged at 400 × g for 3 min. For GM12878 cells, culture medium was removed, and cells were centrifuged at 400 × g for 5 min. For Testis cells, the dissociated cells were centrifuged at 800 × g for 5 min. The cells were processed to fixation, cell lysis, DpnII digestion, and proximal ligation steps following the HiChew protocol.

#### Enzyme digestion and dA-tailing

Cell nuclei were washed twice with 1.1 × rCutSmart buffer (New England Biolabs, Cat. No. B7204S) and resuspended in 200 μL of the same buffer. For digestion, 10 μL of HpyCH4V enzyme (NEB, Cat. No. R0620L) was added, and the reaction was incubated at 37 °C with shaking at 1,000 rpm for 4 h. Aliquots were de-crosslinked with proteinase K at 65 °C for 1 h and analyzed by 1% agarose gel electrophoresis to confirm digestion efficiency.

Following digestion, cell nuclei were pelleted by centrifugation at 500 × g for 3 min, the supernatant was removed, and nuclei were washed twice with washing buffer (1 × PBS, 1 mM EDTA, 1 mM EGTA, 0.1% Triton X-100). Nuclei were then resuspended and counted using a cell counting plate. Approximately 5 × 10^5^ nuclei were transferred to a new 1.5 mL centrifuge tube, and 25 μL of dA-tailing reaction buffer and 10 μL of Klenow fragment (New England Biolabs, NEBNext dA-Tailing Module, Cat. No. E6053L) were added. The reaction volume was adjusted to 250 μL with nuclease-free water, and the mixture was incubated at 37 °C with shaking at 1,000 rpm for 90 min.

After dA-tailing, 200 μL of reaction termination buffer (1 × PBS, 50 mM EDTA, 50 mM EGTA, 0.1% Triton X-100) was added, and the nuclei were centrifuged at 800 × g for 2 min. Nuclei were washed twice with washing buffer, resuspended in 200 μL of the same buffer, and filtered through 20-μm filter. The nuclei concentration was determined using a cell counting plate, and 2 × 10^5^ nuclei were resuspended in 1,165 μL of BSA buffer (1 × PBS, 0.1% Triton X-100, 0.3% BSA).

#### Index adapter ligation

Custom-designed 96 index primers, avoiding the GATC sequence (5’- CGA CCA CCG AGA TGT ACA C—6 bp index—TCG TCG GCA GCG TCA GAT GTG TAT AAG AGA CAG T—3’ and 5’—/phos/CTG TCT CTT ATA CAC ATC TCC GAG CCC ACG AGA C—3’) were used to prepare 50 μM index-Y adapters. Each well of a 96-well plate was loaded with 2 μL of index adapter. The 1,165 μL nuclei solution was well-mixed before loading 11.6 μl of nuclei solution into each well of the 96-well plate. For ligation, 220 μL of 2 × Instant Sticky-End Ligase Master Mix (New England Biolabs, Cat. No. M0370), 352 μL of 5 × Quick Ligase Buffer (New England Biolabs, Cat. No. B6058S), and 132 μL of 1,2-propanediol (Sigma-Aldrich, Cat. No. 398039) were mixed and 6.4 μL of mixture was distributed into each well of the plate containing the nuclei and adapters. The reaction was incubated at 20 °C with shaking at 1,600 r.p.m. for 3 h, with intermittent shaking for 30 s every 5 min.

After ligation, 50 μL of reaction termination buffer was added to each well, mixed, and incubated at room temperature for 15 min. The products from all wells were pooled into a 15 mL centrifuge tube. The combined nuclei were centrifuged at 800 × g for 5 min, washed twice with washing buffer, and resuspended in BSA buffer. Single cells were filtered through a 10-μm filter and counted using a cell counting plate. A total of 930–1,400 nuclei were resuspended in 350 μL of BSA buffer, and 3 μL of the suspension was distributed into each well of a new 96-well plate, with approximately 800—1,200 nuclei in total across all wells.

#### Reverse crosslinking and PCR amplification

For reverse crosslinking, Qiagen protease (Qiagen, Cat. No. 19157) was prepared at 0.1 U/mL in EB buffer, and 3 μL was added to each well. Plates were mixed by shaking, centrifuged briefly, and incubated in a thermocycler at 50 °C for 1 h, 65 °C for 2 h, and 70 °C for 30 min. Next, 0.6 μL of a 10 μM i7 PCR primer (5′-CAA GCA GAA GAC GGC ATA CGA GAT—8 bp index—GTC TCG TGG GCT CGG-3′) was added to each well. The PCR reaction was prepared with 760 μL of 2 × KAPA HiFi ready mix (KAPA Biosystems, Cat. No. KK2602), 65 μL of HiChew-P5 primer (5′-/phos/AAT GAT ACG GCG ACC ACC GAG ATG TAC AC-3′), and 135 μL of nuclease-free water. A total of 9.3 μL of the reaction mix was added to each well of the plate contaning the de-crosslinked DNA, and amplification was performed for 12 −15 cycles on a PCR thermocycle instrument. After amplification, the PCR products from all wells were pooled into a 15 mL centrifuge tube and purified using the QIAGEN PCR purification kit (Qiagen, Cat. No. 28104). Part of the PCR product was processed to library circulation **** and sequencing to produce Dip-C data.

#### Methylation, immunoprecipitation and post-IP PCR amplification

Purified DNA (1 μg) was methylated at GATC sites, performed immunoprecipitation with Anti-m6A antibody and PCR amplified following the HiChew protocol.

### Library circulation and sequencing

Library circularization was performed using the MGIEasy Circulating Kit (MGI, Cat. No. 1000005259) according to the manufacturer’s protocol. The circularized library was processed with the MGISEQ-2000RS High Throughput Sequencing Reagent Kit (PE100) (MGI, Cat. No. 1000012554) to generate DNA nanoballs (DNBs). Sequencing was carried out on the MGISEQ-2000 platform to generate paired-end 100 bp reads.

### Intron-FISH

Specific targeting probes were designed and provided by Spatial FISH Ltd (Shenzhen, China). HEK293T cells were cultured in confocal dish, fixed with 4% paraformaldehyde in PBS and enclosed within a reaction chamber to facilitate subsequent reactions. Following fixation, the samples were dehydrated with methanol and denatured. Hybridization buffer containing the specific targeting probes was then applied to the reaction chamber, and the samples were incubated at 37 °C overnight. After hybridization, the samples were washed three times with PBST, and the targeting probes were ligated in a ligation mix at 25 °C for 3 h. The samples were washed again three times with PBST and subjected to rolling circle amplification using Phi29 DNA polymerase (NEB, Cat. No. M0269L) at 30 °C overnight. Fluorescent detection probes, prepared in hybridization buffer, were subsequently applied to the samples. After hybridization, the samples were dehydrated through an ethanol series and mounted with mounting medium. Images were captured using a OLYMPUS FV3000 Imaging System equipped with a 100 × objective lens (NA = 1.45). The signal dots were decoded to resolve RNA spatial position information. The python package cv2 (v4.10.0) [33] was used to identify the locus of probes and nuclear membrane contour. The smallest Euclidean distance of each identified probe to the nuclei membrane was calculated and summarized by histogram showing probability density distribution.

### Western blot

Western blotting was performed using a Western Blot Kit (Rabbit) with a PVDF membrane (Sangon Biotech, Cat. No. C620393) according to the manufacturer’s instructions. Proteins were transferred from the gel to a PVDF membrane using a wet transfer method. The membrane was incubated with an Anti- *CTCF* antibody (Cell signaling, Cat. No.2899) and Anti-beta Actin antibody (abcam, Cat. No. ab8227) as the primary antibody, followed by an HRP-conjugated goat anti-rabbit IgG H&L (abcam, Cat. No. ab205718) as the secondary antibody. Diaminobenzidine (DAB) reagent was used as the substrate for HRP, and the bands were visualized by direct inspection.

### RNA-seq

Total RNA was extracted from samples using the RNeasy Mini Kit (Invitrogen, Cat. No. 74104) following the manufacturer’s protocol. The RNA-seq library was prepared using the MGIEasy RNA Library Prep Kit (MGI, Cat. No. 1000006383) according to the manufacturer’s instructions. The library was processed with the MGISEQ-2000RS High Throughput Sequencing Reagent Kit (PE100) (MGI, Cat. No. 1000012554) to generate DNA nanoballs (DNBs). Sequencing was carried out on the MGISEQ-2000 platform to generate paired-end 100 bp reads.

### RT-qPCR validation

Total RNA was extracted from samples using the RNeasy Mini Kit (Invitrogen, Cat. No. 74104) following the manufacturer’s protocol. cDNA was synthesized from 1 µg of total RNA using SuperScript™ II Reverse Transcriptase (Invitrogen) and oligo(dT) primers, following the manufacturer’s instructions. qPCR was performed on 96-well plates (Axygen) using the StepOnePlus Real-Time PCR System (Applied Biosystems). Each reaction consisted of 5 μL of TB Green Premix Ex Taq II (Tli RNase H Plus) (Takara, Cat. No. RR82WR), 0.5 μL of 5 μM each primer (CTCF-qPCR-F, 5'-TCC TCT GAC AGT GAA AAT GCT-3’ and CTCF-qPCR-R, 5'-TGT TTG GGC TGG TTG GTT CT-3’ for CTCF-qPCR, GAPDH-qPCR-F, 5'-GAA CGG GAA GCT CAC TGG-3’ and GAPDH-qPCR-R, 5'-GCC TGC TTC ACC ACC TTC T-3’ for GAPDH-qPCR), 1 μL of diluted cDNA, and 3 μL of nuclease-free water, in a total volume of 10 μL. The qPCR cycling conditions were as follows: initial denaturation at 95 °C for 2 min, followed by 40 cycles of 95 °C for 8 s, 55 °C for 30 s, and 72 °C for 30 s. Each qPCR assay was performed in technical triplicate and included three independent biological replicates. Data were analyzed using the 2^-ΔΔCt method for relative quantification.

### Genotyping PCR

Genomic DNA was extracted from HEK293T and HEK293T- *CTCF* ± cells using the PureLink™ Genomic DNA Mini Kit (Invitrogen, no. K182001) following the manufacturer’s instructions. Genotyping PCR was performed using KAPA HiFi readymix in a total reaction volume of 25 μL, containing 1 × PCR mix, 0.5 μM of each primer (CTCF-F, 5'-TCA TGT GCC TAC CAC CCA GC-3’, CTCF-R, 5'-AGG TAG GTT AGG AAA TCT ACC CAG AC-3), and approximately 20 ng of genomic DNA template. The PCR cycling conditions were as follows: initial denaturation at 95 °C for 2 min, followed by 20 cycles of 98 °C for 20 s, 62 °C for 30 s, and 72 °C for 1 min, with a final extension at 72 °C for 5 min. The amplified products were purified with 0.8 volumes of Agencourt AMPure XP Beads and assessed using Agilent 2100 DNA HS Assays. Peak chart was analysed to confirm genotypes.

### ChIP-seq

ChIP-seq was performed following the protocol described by Johnson et al. (2007) [34] with minor modifications. Cells were cross-linked with 1% formaldehyde at room temperature for 10 min to preserve protein-DNA interactions, and the reaction was quenched by the addition of 125 mM glycine. Nuclei were isolated and lysed to release chromatin, which was subsequently sheared to an average fragment size of 200–500 bp using a Bioruptor sonicator (Diagenode). Immunoprecipitation was performed overnight at 4 °C with Anti-CTCF antibody (Cell signaling; Cat. No.2899) and Rabbit Control IgG (Abclonal, Cat. No.AC005). Protein-DNA complexes were captured using magnetic protein A/G beads (Thermo, Cat. No. 88802), followed by reverse cross-linking with proteinase K (QIAGEN, Cat. No. 19133) at 65 °C for 6 h. DNA was extracted using a PCR purification kit (Qiagen, Cat. No. 28004) according to the manufacturer’s instructions. Sequencing libraries were prepared using the MGIEasy Universal DNA Library Prep Kit (MGI, Cat. No. 1000006985) and sequenced on an MGISEQ-2000 platform to generate paired-end 100 bp reads.

### Single-cell RNA Sequencing (scRNA-seq)

Single-cell RNA-seq was performed using the Chromium Single Cell 3' Solution (10 × Genomics) following the manufacturer’s protocol. Briefly, single-cell suspensions of HEK293T cells and HEK293T- *CTCF* ± cells were loaded onto a Chromium Controller to generate nanoliter-scale Gel Bead-in-Emulsions (GEMs), enabling the encapsulation of individual cells with barcoded beads. After cell lysis, RNA molecules were reverse-transcribed to cDNA, incorporating unique molecular identifiers (UMIs) and cell-specific barcodes. The resulting cDNA was amplified and used for library preparation following the Chromium Single Cell 3' Library Construction Kit protocol. Libraries were circularized, prepared DNBs, and sequenced on an MGISEQ-2000 platform to generate paired-end 100 bp reads.

### Preprocessing of bulk HiChew and snHiChew datasets

The raw reads of snHiChew or other benchmarked single-cell chromatin conformation capture technologies were demultiplexed to each cell using an in-house script. This script utilizes multi-thread functions adapted from scHicDemultiplex.py in scHiCExplorer. Single-cell or bulk sample Hi-C paired-end reads were then aligned to the hg19 or mm10 reference genome using HiC-Pro [12] (v.3.1.0, default settings). The nomenclature for HiC-Pro metrics, including terms such as valid pairs and reported pairs, adheres to the conventions established in the HiC-Pro publication. This data was used to generate HiC-Pro filtering and alignment metrics, valid pairs, and contact matrices, which were then corrected with ice_norm. For each chromosome, contact matrices at resolution levels ranging from 5 kb to 2.5 Mb were created for further analysis. To estimate the number of valid cells in the snHiChew data, we employed a barcode rank plot to identify the steep drop-off pattern that distinguishes valid cells from background noise. Specifically, all detected snHiChew barcodes were plotted in descending order based on the number of nonduplicated valid pairs associated with each barcode. The R package kneedle (v1.0.0) was utilized to identify two critical knee points in the data [35]. The first knee point (knee1) was used to differentiate cells that are highly likely to be doublets from valid cells, while the second knee point (knee2) was used to distinguish background noise from valid cells. Barcodes with ranks above knee1 were classified as doublet-prone cells, and those with ranks below knee2 were classified as background noise. Only the data associated with valid cells, which fall between knee1 and knee2, were retained for further analysis. The detailed HiChew quality control tables could be found in Supplementary Tables.

### Comparison of bulk chromatin architecture datasets

We summarized key metrics such as the cis/trans ratio, valid pair ratio, valid pair dup, and others from the HiC-Pro statistical outputs. To clarify, the valid pair ratio was computed by dividing the valid pairs (prior to removing duplication) by the reported pair from HiC-Pro. This effectively shows the enrichment efficiency. The valid pair dup is the duplicated segment of valid pairs as identified by HiC-Pro, which evidences the level of sequencing saturation.

To analyze the contact matrix pattern, we first converted the nonduplicated valid pairs from bulk HiChew and other chromatin conformation capturing methods into mcool files using Cooler (v0.8.2) with the default iterative correction parameter –balance –balance-args '–convergence-policy store_nan’. We then used the R package HiCExperiment (v1.2.0) [36] to perform a paired comparison analysis at different contact map resolutions. This analysis offers insights into Hi-C features at the whole genome, chromosome, topological associated domains (TAD), and chromatin loop levels.

We measured the correlation of contact matrices at chromosomal and TAD levels using genome-wide eigenvector scores (compartment score) and TAD insulation score, calculated by Cooltools (v0.4.1). We then calculated the corresponding correlation using in-house R scripts and Pearson methods. Lastly, the contact distance decay curve was calculated using HiCExplorer (v3.7.2).

### snHiChew collision rate estimation

The snHiChew HEK293T-NIH/3T3 mixture data was processed using a combined genome of hg19 and mm10, using established snHiChew preprocessing techniques. We used nonduplicated valid pairs in the cell rank plot to identify a steep drop-off pattern and pinpoint the empty cell barcodes. For valid cell barcodes, we assigned them to HEK293T if a minimum of 89% of nonduplicated valid pairs were linked to hg19. Similarly, we assigned them to NIH/3T3 if at least 89% of nonduplicated valid pairs were associated with mm10. Remaining valid cell barcodes were classified as doublets.

### Clustering of snHiChew data in HEK293T-NIH/3T3 mixture data

The corresponding pairs related to the annotated HEK293T and NIH/3T3 cell barcodes are combined and marked for grouping. We employed Higashi [37] with standard settings for dimensionality reduction calculations. For UMAP clustering, we used the first 15 principal components of Higashi embeddings with parameters set to “n_neighbors = 5, min_dist = 0.01, metric = 'correlation'”.

### Comparison of single cell chromatin architecture datasets

We compared the performance of snHiChew's chromatin architecture data with published data from Dip-C and snHi-C, well-known single-cell chromatin conformation capture technologies. The datasets were obtained from the National Center for Biotechnology Information (NCBI) Gene Expression Omnibus (GEO) using the codes GSE94489 and GSE146397.

We applied the same preprocessing and HiC-Pro metrics based benchmark procedures to Dip-C, snHi-C, and snHiChew as we previously described. To assess sequencing yield efficiency, we downsampled raw reads to various levels: 15.625 k, 31.25 k, 62.5 k, 125 k, 250 k, 500 k, 1 M, 2 M, 4 M. We only included cells with read numbers that exceeded these thresholds.

We then calculated the number of non-duplicated valid pairs per read for each threshold, fitting the data points to a saturation curve using the model *Y* = *B max* * *X*/(*Kd* + *X*). Finally, we estimated the read number for sequencing saturation for each single-cell chromatin conformation capture technology.

The false positive rate was assessed by looking at the unexpected interactions between mitochondrial DNA and nuclear DNA. We computed the false positive rates for each unique valid pair identified by HiC-Pro in every valid cell of the snHiChew and Dip-C datasets, using the method described in Trac-looping [38].

### Identifying TAD, melting, and concretion states

Valid snHiChew cells were first downsampled to the same number of non-duplicate valid pairs and converted to mcools using Cooltools (v0.8.2) with the default iterative correction parameter –balance –balance-args '–convergence-policy store_nan'. TAD boundary identification was performed using hicFindTADs from HiCExplorer (v3.7.2) with multiple window sizes and –thresholdComparisons set to 0.01 for TADs and 0.05 for TADs. The insulation score for each valid snHiChew single cell was calculated using hicFindTADs with a resolution of 50,000, –minDepth of 200,000, –maxDepth of 750,000, –step of 50,000, –thresholdComparisons of 0.05, –delta of 0.01, and –correctForMultipleTesting set to FDR. The TAD-separation score generated in the bedgraph was recognized as the single-cell insulation score.

To identify the melting and concretion states of the single cells, hierarchical clustering (ward.D or ward.D2) was performed on the scaled single-cell insulation scores for each 50 k genomic bin in the region of interest. The cutree function in R was used to define two clusters with k = 2. The cell cluster with the higher median single-cell insulation score was identified as the concretion state cluster, while the cluster with the lower median score was identified as the melting state cluster.

After obtaining the two cell clusters in a given region of interest, the MELTRON [19] pipeline was adapted to define the melting state of each genomic region of interest. In brief, melting states were calculated for HEK293T B compartment genes longer than 300 kb on the hg19 genome as the genomic region of interest. The cumulative probability distribution of the single-cell insulation scores for the melting and concretion clusters was calculated and compared using the Kolmogorov–Smirnov test. The degree of melting was defined by the maximum distance (D) between the two distributions of the melting and concretion cell clusters, with a significance level of *p* < 0.00001. For visualization, the median insulation score values for each 50 k genomic bin of each cluster were used to calculate the cumulative probability distribution in the genomic area of chr7:145 M-155 M.

### In silico cell phasing over the cell cycle

We carried out cell-cycle analysis following the method detailed in a prior study [39]. In short, we used the HEK293T 2-phase Repli-seq dataset (4DNESSV33VOL, 4DNESH4XLJCW) obtained from the 4D Nucleome Data Portal to label the early/late repli-score ratio for each cell. A higher early/late repli-score ratio indicates that the cell is nearer to the early S-phase of the cell cycle.

### Three-dimensional genome modeling

We utilized Chrom3D (v1.0.2) [40] to generate three-dimensional structural genome models. The model setup files were created from the HiC-Pro raw contact matrix of the whole hg19 genome with a 50 kb bin. The exemplary valid cells were chosen to symbolize the melting and concretion phases. We adhered to the Chrom3D protocol for the modeling process, which can be found at https://github.com/Chrom3D/pipeline. In the model setup file, three regions of interest (ROI) were distinctly color-labeled. The models were generated with the following settings: Chrom3D -n 3,000,000 -r 5.0 -y 0.15 -l 10,000 [Model Setup File] > [Output file (CMM)]. Snapshots were taken from the models to demonstrate the stable energy-minimized structures in the ROI. The distance to the nuclear membrane was estimated by calculating the minimum 3D Euclidean distance from the TAD beads within ROI to the cLAD regions of HEK293T obtained from GSE156150 bulk dataset.

### ChIP-seq analysis

Adapter sequences and low-quality bases were removed using Fastp (v0.23.2) [41] with default settings. Clean reads were aligned to the hg19 reference genome using Bowtie2 (v2.5.1) [42] with the –very-sensitive, –end-to-end, –phred33, -I 10, and -X 700 options. Aligned reads were sorted using Samtools (v1.6) [43] and duplicates were marked and removed using Picard (v2.18.29) (http://broadinstitute.github.io/picard/). Peaks of *CTCF* IP samples were called using MACS2 (v2.1.4) [44] with the corresponding input as control. Differential peak analysis was performed using DiffBind (v3.14.0) [45] in R (v4.4.0). Two biological replicates of IP and INPUT for WT and *CTCF* ± cell lines were used.

### Bulk RNA-seq analysis

Adapter sequences and low-quality bases were removed using Fastp (v0.23.2) with default parameters. The reference genome and annotation files were prepared for RSEM (v1.3.3) [46] using Bowtie2 (v2.5.1). The reference genome Fasta and gene annotation GTF file were downloaded from the UCSC hg19 genome. Trimmed reads were aligned to the reference genome, and gene and isoform abundances were quantified using RSEM with Bowtie2. RSEM expected count was used for downstream gene and transcript analysis.

### scRNA-seq analysis

scRNA-seq analysis follows our previous publication [23]. In brief, raw sequencing data were processed using the CellRanger pipeline (v3.1.0, 10X Genomics) [47] to generate gene expression matrices. The output from CellRanger was further processed using Seurat (v3) [48] for quality control, filtering, normalization and scaling using default parameters.

### Statistics

We conducted the two-sided t-test on most of the parametric data, which followed a normal (or log-normal) distribution. For the same or different datasets, we also performed a Pearson correlation analysis. We used the two-sided Wilcoxon rank test for non-parametric data or data that was not normally distributed.

## Supplementary Information

Additional file 1: Supplementary Fig. S1-S11.

Additional file 2: Method supplement note.

Additional file 3: Table S1. Metadata of the bulkHiChew HEK293T library.

Additional file 4: Table S2. Metadata of the single cells detected in the snHiChew HEK293T-NIH3T3 library.

Additional file 5: Table S3. Metadata of the single cells detected in the snHiChew HEK293T 200 cell library.

Additional file 6: Table S4. Metadata of the single cells detected in the snHiChew GM12878 library.

Additional file 7: Table S5. Metadata of the single cells detected in the snHiChew HEK293T 1200 cell library.

Additional file 8: Table S6. Metadata of the single cells detected in the snHiChew HEK293T 1200 cell library.

Additional file 9: Table S7. Metadata of the *CTCF* ChIP-seq for HEK293T WT and *CTCF* knockdown cell library.

Additional file 10: Table S8. Metadata of the single cells detected in the snHiChew HEK293T *CTCF* kd cell library.

Additional file 11: Table S9. Metadata of the single cells detected in the snHiChew HEK293T tripto cell library.

Additional file 12: Table S10. Metadata of the single cells detected in the snHiChew testis library.

Additional file 13: Table S11. List of primers.

Additional file 14: Table S12. Barcoded adapter primers_plate.

Additional file 15: Table S13. Barcoded PCR primers.

Additional file 16. Uncropped gels and blots images.

## Data Availability

The data were stored at NCBI BioProject PRJNA1109567 [49]. The data processed in this study have been submitted to the Gene Expression Omnibus with the accession code GSE284602 [50]. Other public datasets used in this study were downloaded from NCBI GEO with the following accession numbers: ChIP-seq (*CTCF* ENCSR135CRI H3K4me3 ENCSR000DTU; H3K27ac ENCSR000FCH) [51], processed data of HEK293T RNA-seq(GSE85161) [52], mESC snHi-C (GSE94489) [53], mESC Dip-C (GSE146397) [54], HEK293T in situ Hi-C (GSE143465) [55], GM12878 Hi-C (GSE63525) [56], GM12878 LiMCA (GSE240114) [57], GM12878 sciHiC (4DNEXE63LNM5) [58], HEK293T Repli-seq (4DNBSR3CBAW3) [59], HEK293T bulk DamID (GSE156150) [60]. The source data are provided with this paper. Custom scripts used in this study are available from (https://github.com/genometube/snHiChew) [61]. Scripts were also uploaded to https://doi.org/10.5281/zenodo.19042390 [62]. MIT license is assigned to the Github and Zenodo scripts.
