# Supplementary figures and images for: Highly efficient chromatin conformation capture with post-enrichment in single cells by HiChew
Source: Genome Biol. 2026 Apr 27;27:127. doi: 10.1186/s13059-026-04059-1 (PMC13112749; doi:10.1186/s13059-026-04059-1)

Fig. S5a

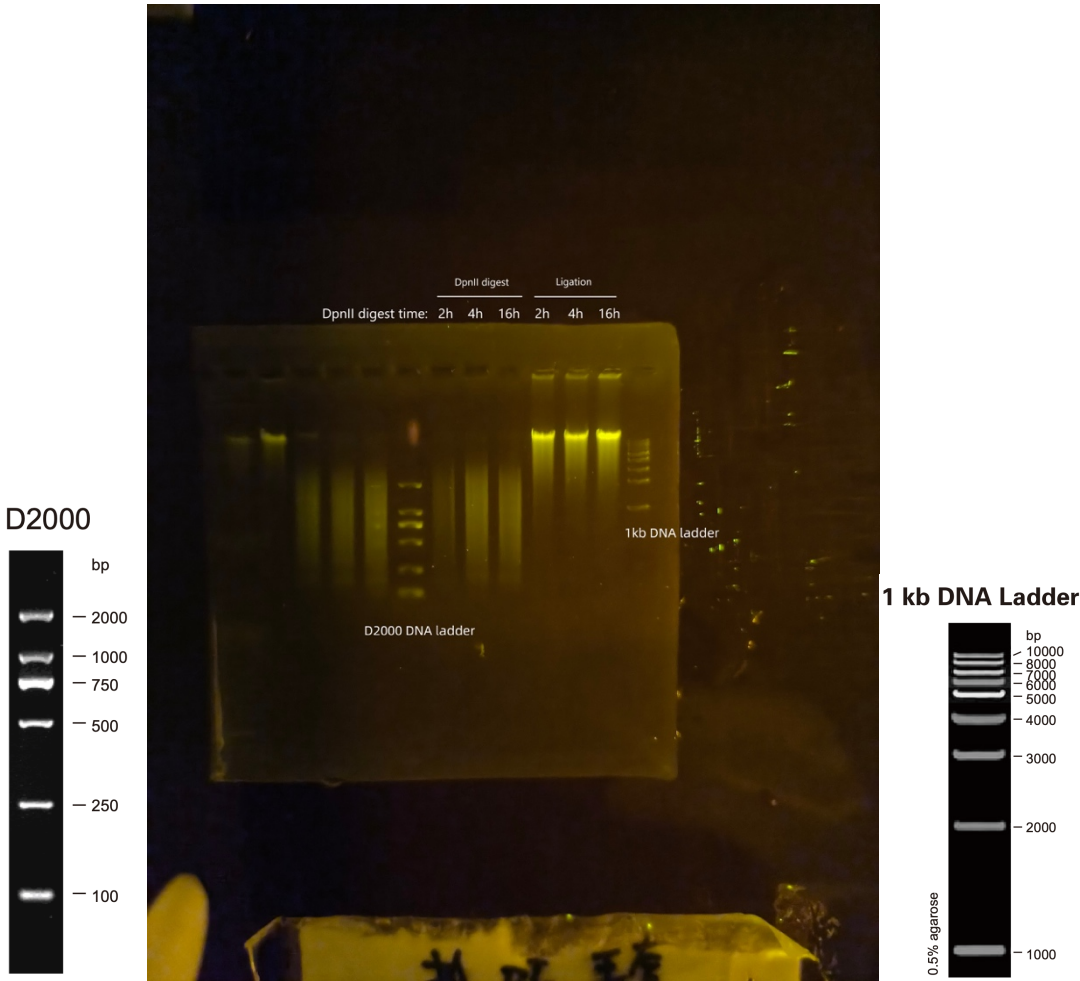

Fig. S5b

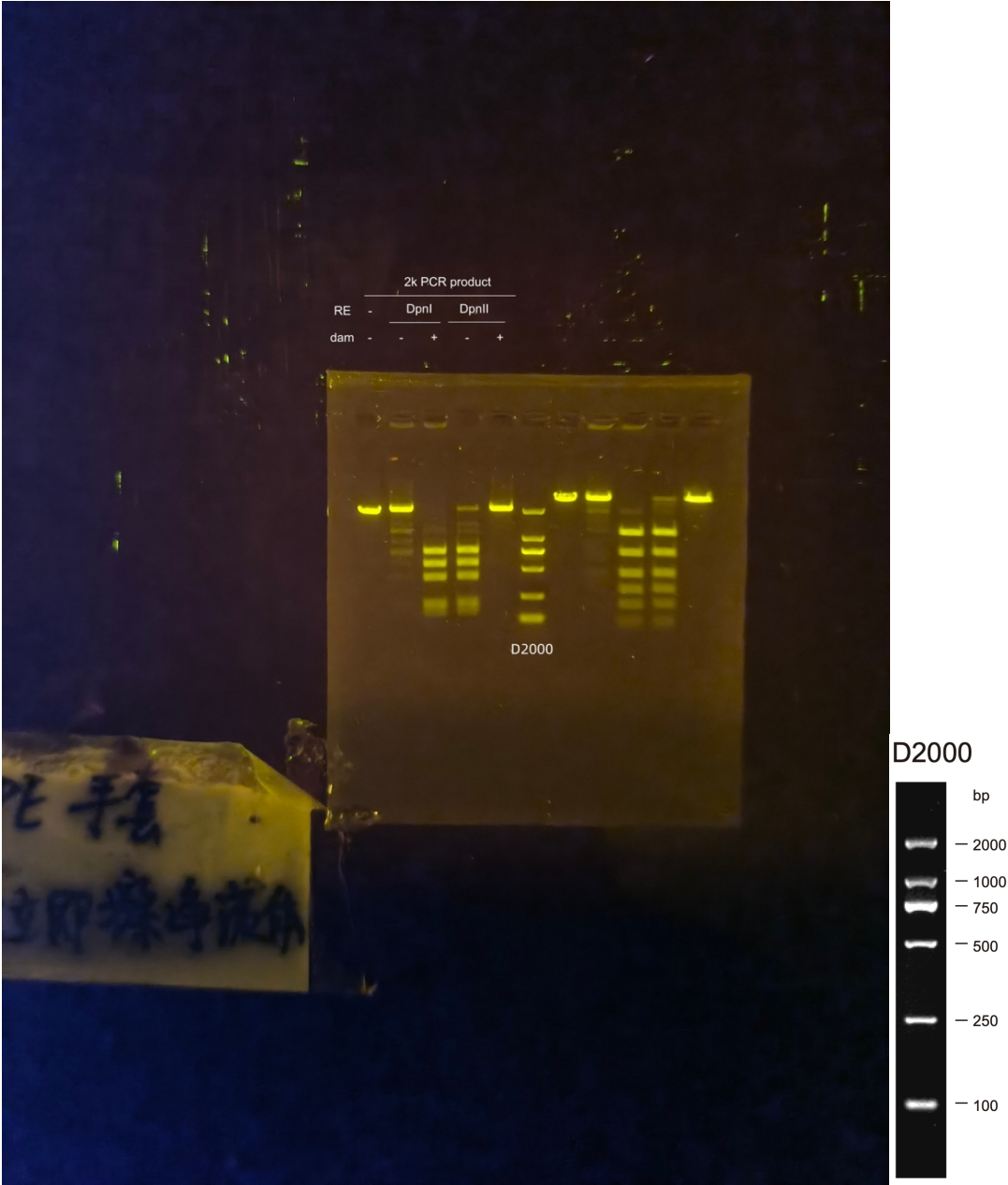

Fig.S5i

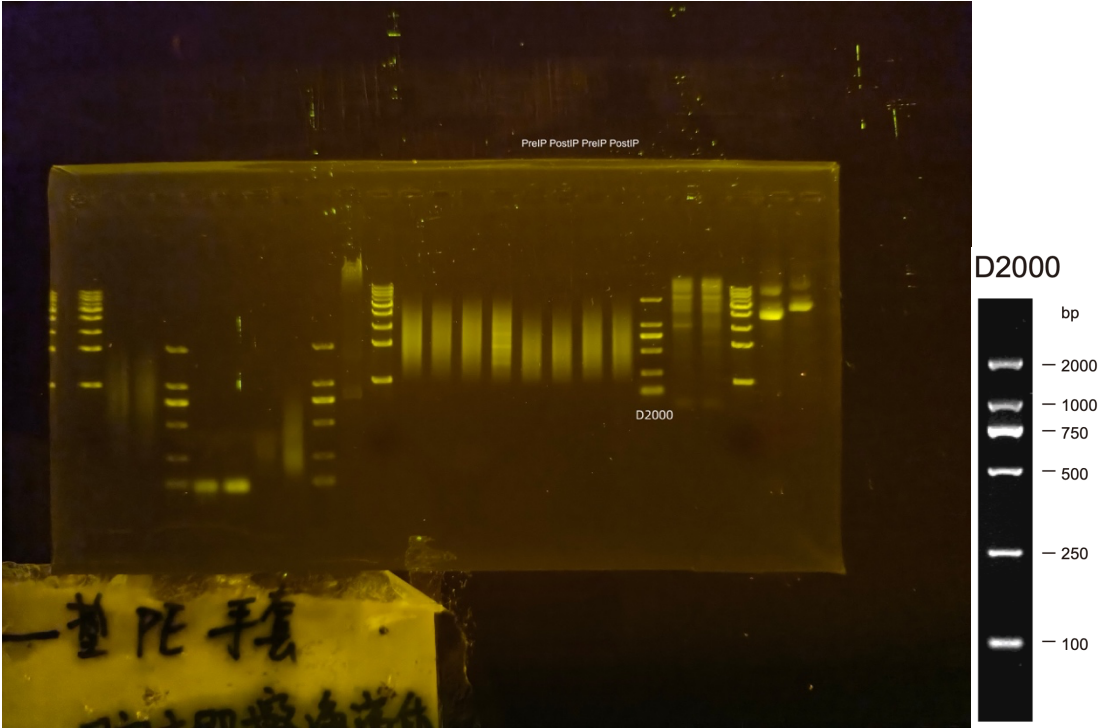

Fig. S9b

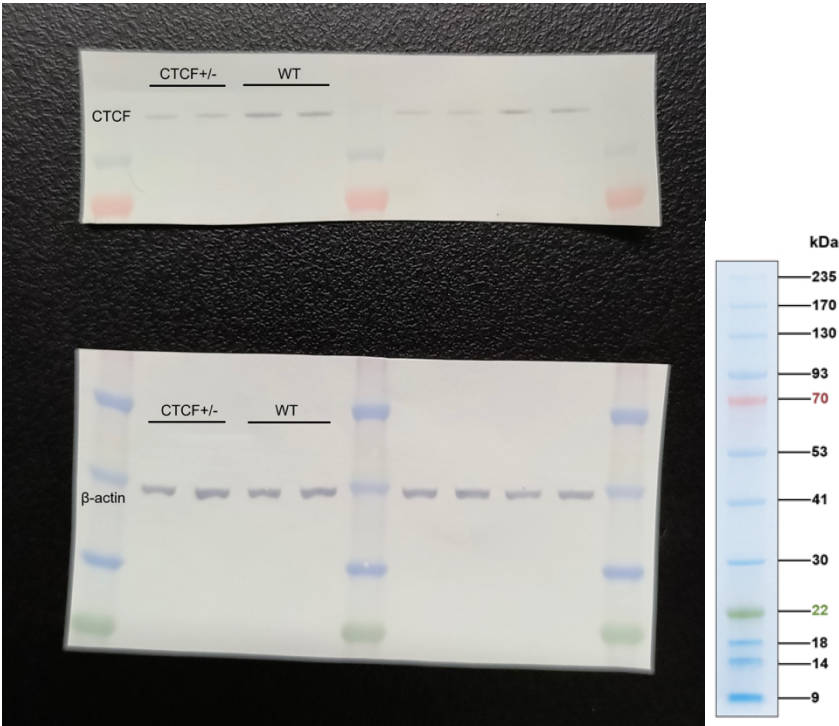

Fig. S10a

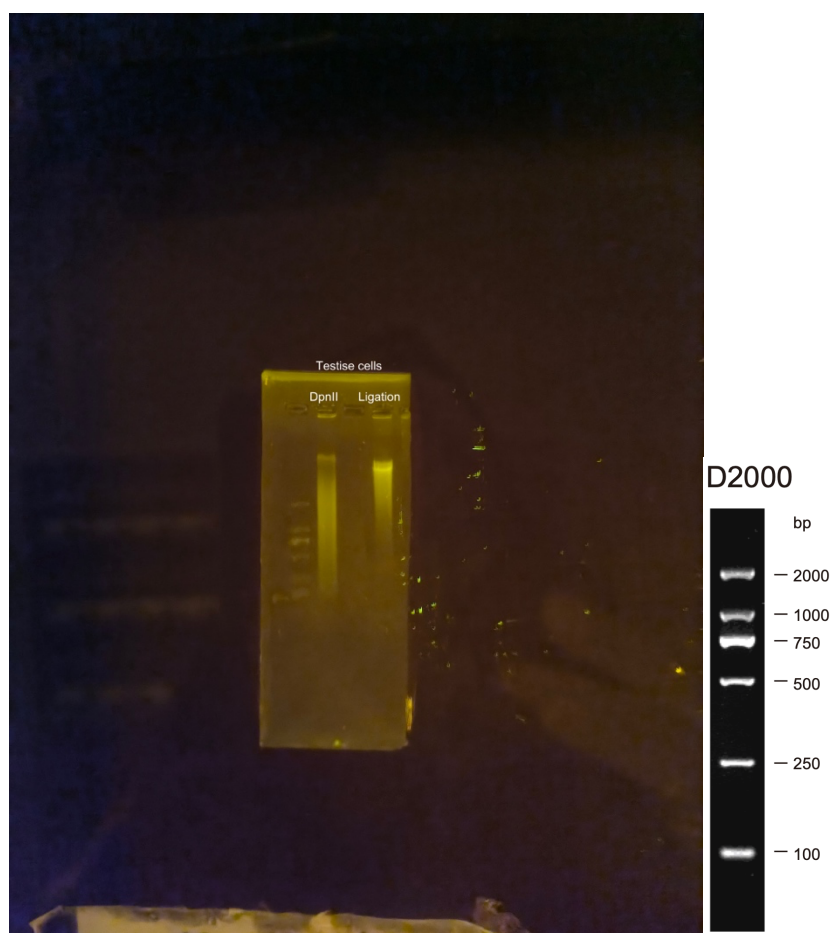

Supplement: Supplementary file 7 — Additional file 7: Table S5. Metadata of the single cells detected in the snHiChew HEK293T 1200 cell library. [file 13059_2026_4059_MOESM7_ESM.pdf]
