# Supplementary material for: Highly efficient chromatin conformation capture with post-enrichment in single cells by HiChew
Source: Genome Biol. 2026 Apr 27;27:127. doi: 10.1186/s13059-026-04059-1 (PMC13112749; doi:10.1186/s13059-026-04059-1)
Supplement: Supplementary file 8 — Additional file 8: Table S6. Metadata of the single cells detected in the snHiChew HEK293T 1200 cell library. [file 13059_2026_4059_MOESM8_ESM.pdf]

Fig. S1

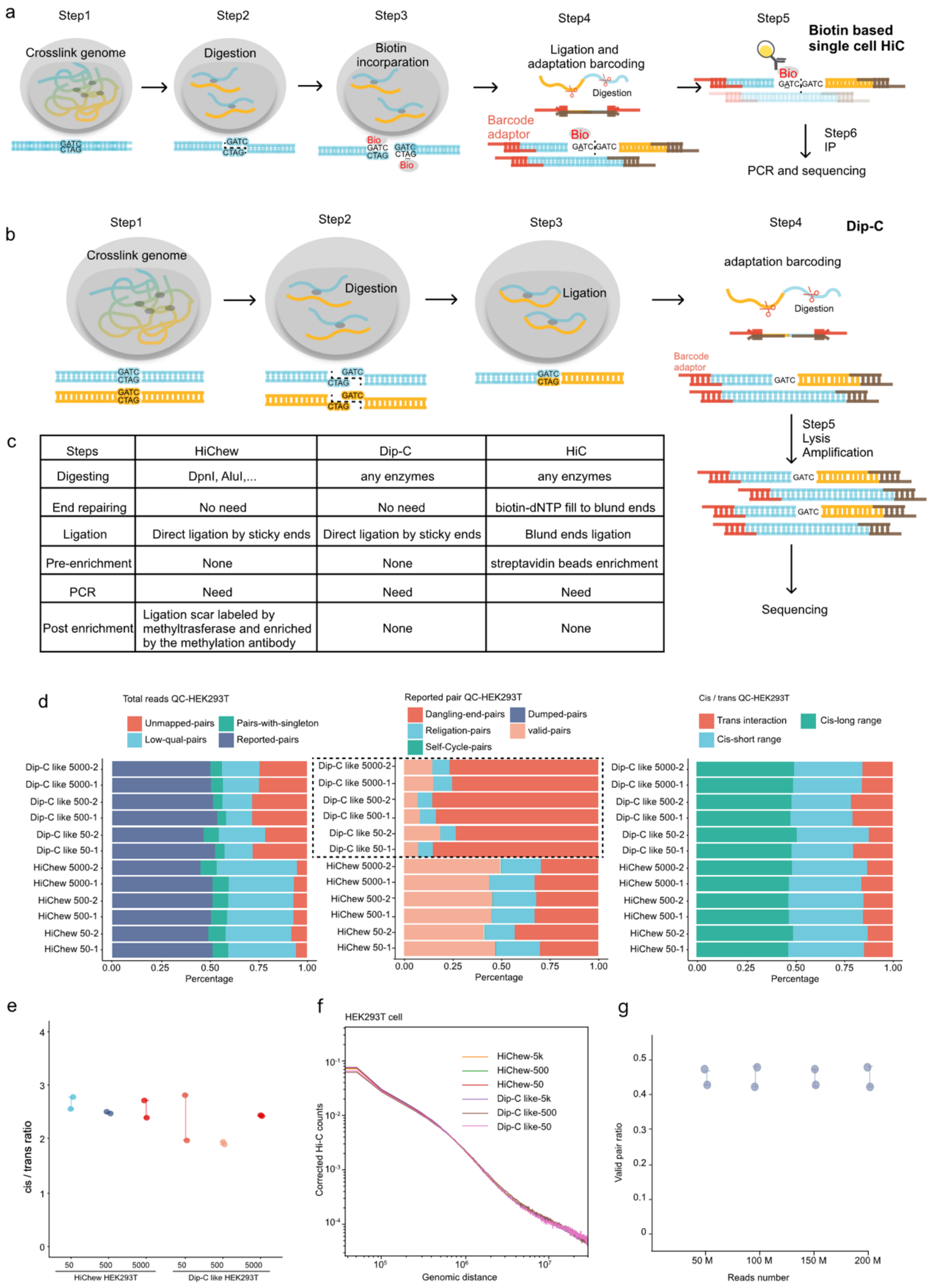

Steps	HiChew	Dip-C	HiC
Digesting	DpnI, AluI,...	any enzymes	any enzymes
End repairing	No need	No need	biotin-dNTP fill to blund ends
Ligation	Direct ligation by sticky ends	Direct ligation by sticky ends	Blund ends ligation
Pre-enrichment	None	None	streptavidin beads enrichment
PCR	Need	Need	Need
Post enrichment	Ligation scar labeled by methyltrasferase and enriched by the methylation antibody	None	None

Fig. S1: Methodological comparison and assessment of HiChew, Dip-C, and Hi-C protocols. This figure presents a detailed technical comparison of two advanced chromatin analysis methods: a, Biotin based Hi-C Protocol: A six-stage process incorporating biotin-based scHi-C technology. The workflow encompasses genome crosslinking for three-dimensional structure preservation, enzymatic DNA digestion, biotin-mediated junction marking, integrated ligation and barcode adaptation, followed by IP enrichment and sequencing procedures. b, Dip-C Protocol: A five-stage methodology comprising genome crosslinking, DNA digestion, ligation processes, barcode adaptation, and concluding with cellular lysis and amplification prior to sequencing analysis. c, Comparative analysis of procedural variations between methodologies. d, Data quality metrics evaluation comparing HiChew and Dip-C methodologies. Left panel: Stacked bar visualization illustrating proportional distribution of unmapped pairs, singleton pairs, and reported pairs across varying cell inputs (50, 500, and 5000 cells). Middle panel: Comprehensive breakdown of reported pair categories, including dangling-end, dumped, religation, self-cycle, and valid pairs, expressed as percentages. Right panel: Aggregate reported cis-long, cis-short, trans pairs per experimental condition. e, Analysis of cis to trans ratios across 50/500/5000 cell inputs in both methodologies. The error bar represents standard error (SE). f, Genomic distance-dependent contact probability analysis across protocols and cell inputs, demonstrating consistent chromatin organization capture across experimental conditions. g, A scatter plot displaying valid pair ratios across different sequencing depths (50M, 100M, 150M, 200M reads) for the HiChew methodology.

Fig. S2

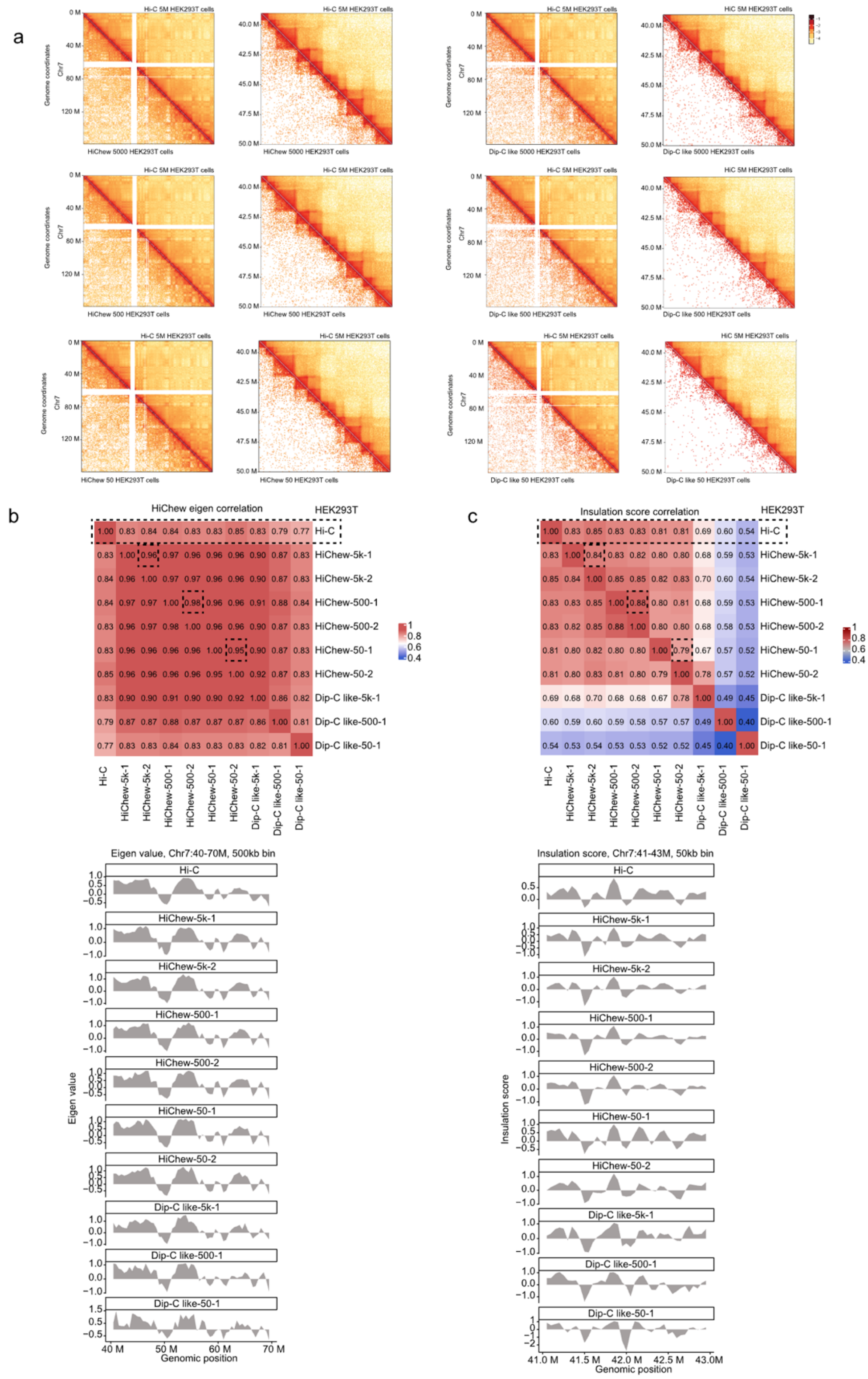
a
b
HiChew eigen correlation
HEK293T
c
Insulation score correlation
HEK293T
Eigen value, Chr7:40-70M, 500kb bin
Eigen value
Genomic position
Insulation score, Chr7:41-43M, 50kb bin
Insulation score
Genomic position

Fig. S2: Comparison of chromatin interaction patterns and correlation analysis across different methods and cell inputs (HEK293T). a, Contact matrices showing chromatin interactions at different resolutions comparing Hi-C (5M cells) with HiChew and Dip-C methods using varying cell inputs (5000, 500 and 50 cells). Left panels show broader genomic regions (AB compartment) while right panels display zoomed-in views of specific chromosomal domains (TADs). Color intensity represents interaction frequency, with warmer colors indicating higher interaction frequencies. **b**, Correlation analysis of eigenvector values across different experimental conditions. The heatmap shows pairwise Pearson correlations between different methods and cell inputs. Bottom panel displays eigenvalue tracks across chr7: 40-70M demonstrating the consistency of compartment signals across different conditions. Color scale ranges from blue (negative correlation) to red (positive correlation). **c**, Insulation score correlation analysis comparing different experimental conditions. Top: correlation heatmap showing the relationship between insulation scores across methods and cell inputs. Bottom: insulation score tracks for chr7: 41-43M region showing the preservation of domain boundary signals.

Fig. S3

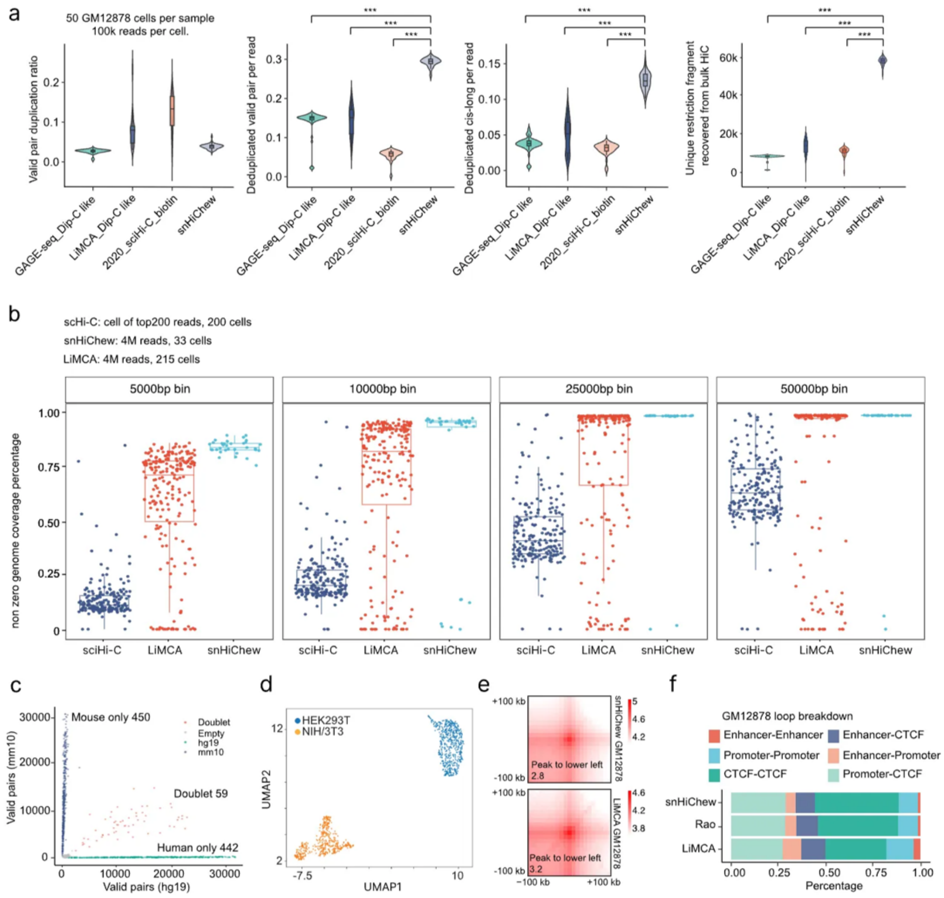

a
50 GM12878 cells per sample
100k reads per cell.
Valid pair duplication ratio
Deduplicated valid pair per read
Deduplicated cis-long per read
Unique restriction fragment recovered from bulk HiC
GAGE-seq_Dip-C like
LiMCA_Dip-C like
2020_sciHi-C_biotin
snHiChew
***
b
scHi-C: cell of top200 reads, 200 cells
snHiChew: 4M reads, 33 cells
LiMCA: 4M reads, 215 cells
5000bp bin
10000bp bin
25000bp bin
50000bp bin
non zero genome coverage percentage
sciHi-C
LiMCA
snHiChew
c
Mouse only 450
Doublet
Empty
hg19
mm10
Doublet 59
Human only 442
Valid pairs (mm10)
Valid pairs (hg19)
d
HEK293T
NIH/3T3
UMAP2
UMAP1
e
+100 kb
-100 kb
Peak to lower left 2.8
snHiChew GM12878
Peak to lower left 3.2
LiMCA GM12878
f
GM12878 loop breakdown
Enhancer-Enhancer
Enhancer-CTCF
Promoter-Promoter
Enhancer-Promoter
CTCF-CTCF
Promoter-CTCF
snHiChew
Rao
LiMCA
Percentage

Fig. S3: Methodological assessment of single cell chromatin conformation capture techniques. a, Systematic evaluation of performance metrics across chromatin capture methodologies. Violin plots illustrate the distribution of critical quality parameters: valid pair duplication ratios, deduplicated valid pair per read,deduplicated cis-long contact per read ratios, and recovered contacts from Hi-C across protocols, encompassing GAGE-seq, LiMCA, scHi-C and snHiChew approaches. Two-sided Wilcoxon signed-rank test was used, *** indicates $P < 0.001$. **b**, Analysis of non-zero bin coverage at multiple resolutions. Box plots present a comparative analysis of non-zero coverage bin percentages among scHi-C, LiMCA, and snHiChew methodologies across various resolutions (5,000, 10,000, 25,000, and 50,000 bp bins). Cells were standardized to 4M reads per cell for snHiChew and LiMCA, with the exception of sciHi-C due to insufficient read depth per cell. **c**, Rigorous quality control assessment of cell demultiplexing, presenting the distribution of 1,200 processed cells post-doublet removal. The scatter plot illustrates Log2(hg19) versus Log2(mm10) read distributions, with distinct population categorization: doublets (red), empty droplets (gray), hg19-specific cells (blue), and mm10-specific cells (green). Key statistical parameters include doublet frequency (0.052) and demultiplexing threshold (259). **d**, Cell-type differentiation analysis demonstrating distinct clustering of HEK293T (blue) and 3T3 (orange) populations based on chromatin interaction signatures. **e**, Aggregate peak analysis (APA) plots comparing loop strength of GM12878 pseudo bulk contacts between LiMCA and snHiChew methods, with enrichment scores to the lower left shown below each plot. **f**, Stacked bar chart illustrating the proportion of different functional categories of chromatin loops—including Enhancer-Promoter, Enhancer-*CTCF*, and Promoter-*CTCF* interactions—called from the gold-standard Hi-C, snHiChew (pseudo bulk) and LiMCA (pseudo bulk) methods.

Fig. S4

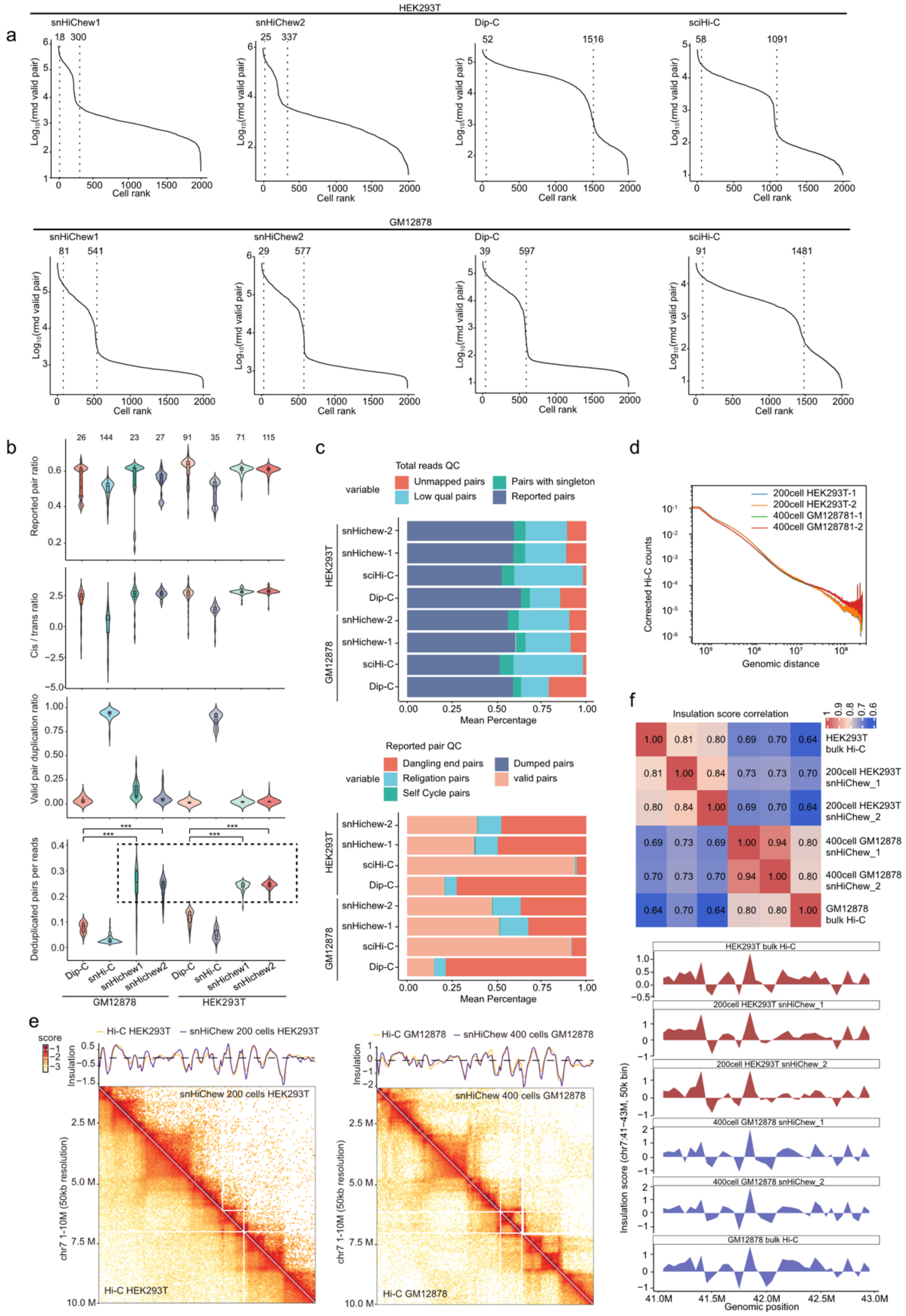

a
HEK293T
snHiChew1
snHiChew2
Dip-C
sciHi-C
GM12878
Log10(rmd valid pair)
Cell rank
b
Reported pair ratio
Cis / trans ratio
Valid pair duplication ratio
Deduplicated pairs per reads
Dip-C
snHi-C
snHichew1
snHichew2
GM12878
HEK293T
c
Total reads QC
variable
Unmapped pairs
Low qual pairs
Pairs with singleton
Reported pairs
Mean Percentage
Reported pair QC
Dangling end pairs
Religation pairs
Self Cycle pairs
Dumped pairs
valid pairs
d
200cell HEK293T-1
200cell HEK293T-2
400cell GM128781-1
400cell GM128781-2
Corrected Hi-C counts
Genomic distance
e
Hi-C HEK293T
snHiChew 200 cells HEK293T
Hi-C GM12878
snHiChew 400 cells GM12878
score
Insulation
chr7 1-10M (50kb resolution)
f
Insulation score correlation
HEK293T bulk Hi-C
200cell HEK293T snHiChew_1
200cell HEK293T snHiChew_2
400cell GM12878 snHiChew_1
400cell GM12878 snHiChew_2
GM12878 bulk Hi-C
Insulation score (chr7:41~43M, 50k bin)
Genomic position

Fig. S4: Quality control and analysis of snHiChew performance metrics in GM12878 and HEK293T cell lines. a, Quantitative assessment of cellular performance via rank distribution plots, illustrating valid pair frequencies across methodologies (snHiChew, Dip-C, and scHi-C) in GM12878 and HEK293T cell lines. Cell rank is plotted against logarithmic valid pair counts, with selection thresholds indicated by dashed lines. **b**, Systematic evaluation of methodological quality parameters. Distribution analysis via violin plots examines four critical metrics: reported pair ratio, cis/trans ratio, valid pair duplication ratio, and deduplicated pairs per reads, across protocols in both cell lines. Two-sided Wilcoxon signed-rank test was used, *** indicates $P < 0.001$. **c**, Rigorous sequencing quality evaluation. Upper panels present proportional distributions of read classifications (unmapped, low quality, singleton, and reported pairs) per methodology. Lower panels detail the composition of reported pairs (dangling end, dumped, religation, self-cycle, and valid pairs) across methodologies and cell lines. **d**, Distance-dependent interaction decay analysis performed by snHiChew for HEK293T and GM12878 cells. **e**, Comparison of chromatin organization features between different experimental conditions and methods. Contact matrices and insulation score comparisons. Left: Hi-C contact matrix of HEK293T cells (2.5M-10.0M region) compared with snHiChew data from 200 cells, with corresponding insulation score tracks shown above. Right: Similar comparison for GM12878 cells showing the bulk Hi-C contact matrix compared with snHiChew data from 400 cells, with corresponding insulation score tracks. Contact matrices are displayed using a red color gradient, where darker red indicates higher interaction frequency. **f**, Correlation matrix showing insulation score correlations between HEK293 scHi-C, 200-cell HEK293 snHiChew replicates, 400-cell GM12878 snHiChew replicates, and GM12878 scHi-C samples. The color scale represents correlation coefficients from blue (low) to red (high). Below the correlation matrix, insulation score tracks are shown for the chr7:41-43M region across all experimental conditions, demonstrating the consistency of domain boundary detection. Red tracks represent HEK293 cell samples, while blue tracks represent GM12878 samples.

Fig. S5

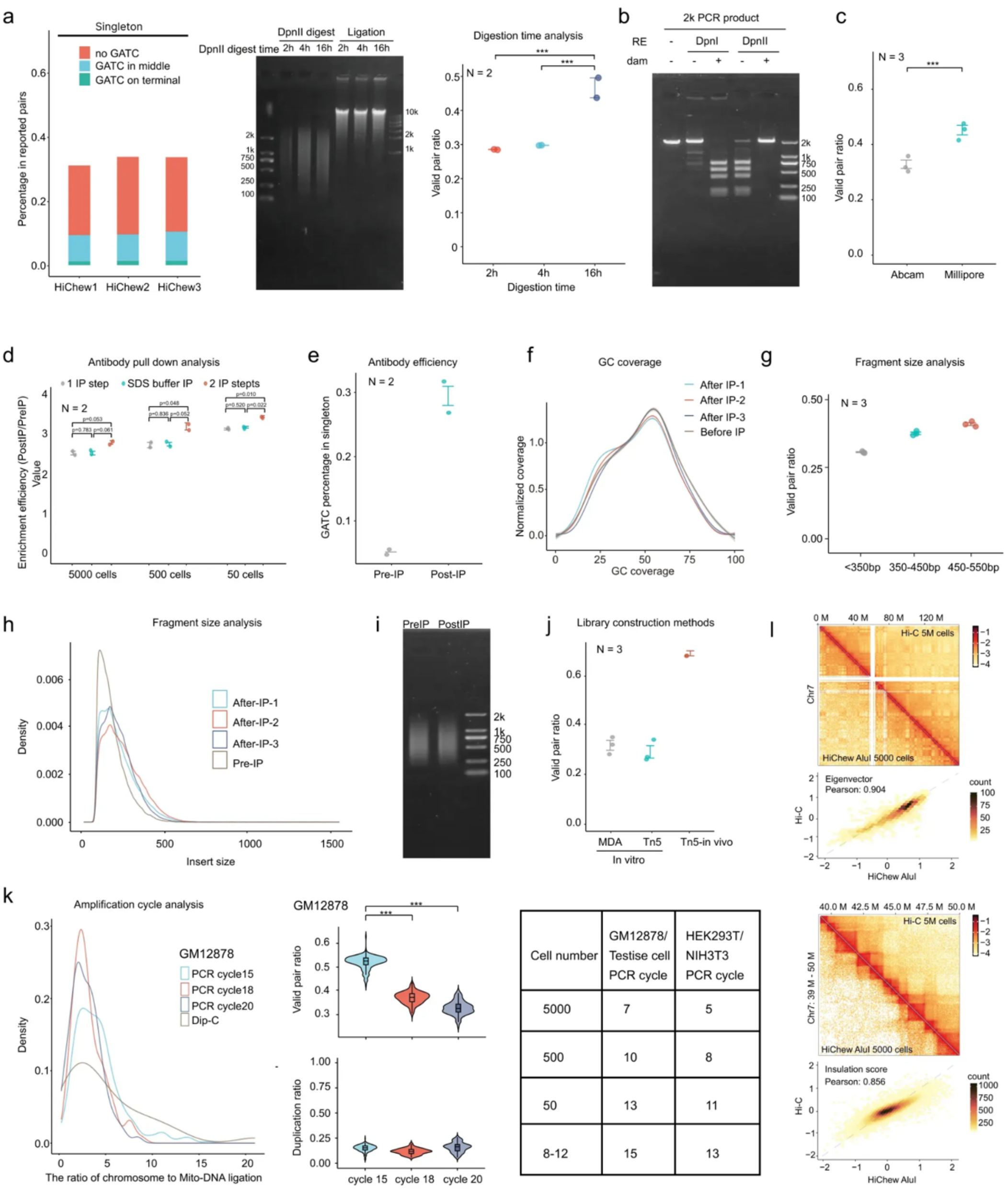

Cell number	GM12878/ Testise cell PCR cycle	HEK293T/ NIH3T3 PCR cycle
5000	7	5
500	10	8
50	13	11
8-12	15	13

Fig. S5: Technical validation and optimization of HiChew. a, Analysis of sequence composition, examining the distribution of singleton reads containing GATC in central positions, GTAC at terminal positions, and sequences without GATC. Electrophoretic analysis reveals the progression of digestion and ligation processes, with box plots demonstrating valid pair ratios throughout digestion (N=2, *** indicates p-value < 0.01). P values are from two-sided two-sample t-tests. **b**, Evaluation of Dam methylation efficiency through DNA fragment analysis. Following Dam labeling and DpnI digestion for m6A detection, samples underwent an assessment to verify GATC methylation status. DpnII digestion served as experimental control, with non-treated genome samples demonstrating complete digestion and Dam-labeled samples confirming methylation-based protection. **c**, Evaluation of antibody efficacy comparing Millipore and Abcam products through valid pair ratio analysis (N=3, p-value = 0.0061). P values are from two-sided two-sample t-tests. **d**, Comprehensive assessment of enrichment strategies across multiple cell inputs (5000, 500, and 50 cells), evaluating single IP enrichment, double IP enrichment, and SDS stringent washing protocols. P values are from two-sided two-sample t-tests. **e**, Assessment of GATC frequency in singleton reads before and after IP enrichment. **f**, Analysis of GC content distribution between pre-IP samples and post-IP replicates. **g**, Investigation of fragment size effects on valid pair ratios across three distinct size ranges (500bp, 400bp, 300bp). **h**, Detailed analysis of fragment size profiles comparing pre-IP and post-IP enrichment samples across three experimental replicates. **i**, Electrophoretic verification of DNA fragment distributions before and after IP enrichment. **j**, Evaluation of library construction efficiency across multiple stages (MDA, Tn5, and Ligation) using valid pair metrics. **k**, Optimization of PCR parameters through analysis of chromosomal to mitochondrial DNA ratios and valid pair metrics across varying amplification cycles (15, 18, and 20 cycles). Two-sided Wilcoxon signed-rank test was used, *** indicates $P < 0.001$. **l**, Comparative evaluation of Hi-C (5M cells) and HiChew AluI (5000 cells) methodologies, employing AluI digestion and Alu methyltransferase for junction labeling, with associated Pearson correlation coefficient metrics (Eigenvector: 0.904, Insulation score: 0.856). The error bar in this fig panel represents the standard error (SE).

Fig. S6

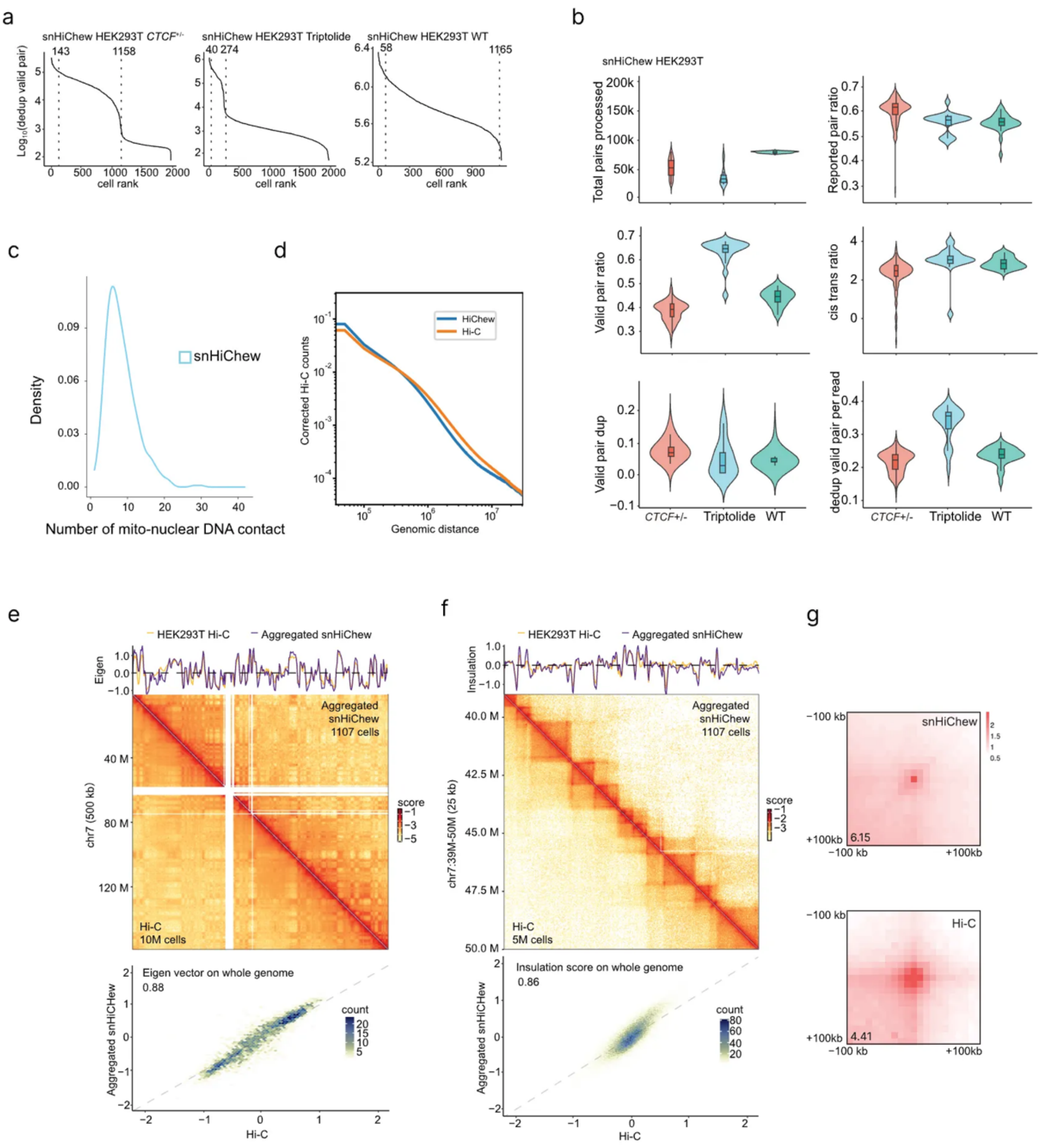
a
snHiChew HEK293T CTCF+/-
snHiChew HEK293T Triptolide
snHiChew HEK293T WT
Log10(dedup valid pair)
cell rank
b
snHiChew HEK293T
Total pairs processed
Reported pair ratio
Valid pair ratio
cis trans ratio
Valid pair dup
dedup valid pair per read
CTCF+/-
Triptolide
WT
c
snHiChew
Density
Number of mito-nuclear DNA contact
d
HiChew
Hi-C
Corrected Hi-C counts
Genomic distance
e
HEK293T Hi-C
Aggregated snHiChew
Eigen
Aggregated snHiChew 1107 cells
Hi-C 10M cells
chr7 (500 kb)
score
Eigen vector on whole genome
0.88
count
Aggregated snHiChew
Hi-C
f
HEK293T Hi-C
Aggregated snHiChew
Insulation
Aggregated snHiChew 1107 cells
Hi-C 5M cells
chr7:39M-50M (25 kb)
score
Insulation score on whole genome
0.86
count
Aggregated snHiChew
Hi-C
g
snHiChew
6.15
Hi-C
4.41
−100 kb
+100kb

Fig. S6: Evaluation of single-cell chromatin organization in HEK293T cells using snHiChew. **a**, Quantitative assessment of cellular valid pair distribution, presenting valid pair analysis across HEK293T cells (*CTCF*$^{+/-}$and treated conditions) post-doublet removal, yielding 250-1015 cells suitable for analysis. **b**, Comprehensive quality assessment across experimental conditions, with left panel illustrating total processed pairs, valid pair ratios, and duplication metrics, while right panel presents reported pair ratios, cis-trans distributions, and unique valid pair/read metrics across cell populations (Hek293T-*CTCF*$^{+/-}$, n=208, Hek293T-Tripotolide, n=20, HEK293T-WT, n=34). **c**, Quantitative evaluation of mitochondrial-nuclear DNA interaction frequencies, establishing baseline false positive rates in snHiChew methodology. **d**, Genomic distance-dependent contact probability analysis, demonstrating concordance between Hi-C (blue) and snHiChew (orange) methodologies. **e**, Systematic comparison of chromatin architectural features between Hi-C and aggregated HEK293T WT snHiChew datasets. Upper panel: Contact matrices at 500kb resolution illustrating chromosome-wide interactions. Lower panel: Eigenvector correlation analysis between methodologies (Pearson correlation coefficient: 0.894). **f**, Fine-scale chromatin organization assessment. Left: High-resolution contact matrices (10Mb region, 50kb resolution). Right: Statistical comparison of insulation metrics between methodologies (Pearson correlation coefficient: 0.855). **g**, Quantitative loop strength evaluation via Aggregate Peak Analysis (APA), with comparative metrics (snHiChew: 6.15, Hi-C: 4.41).

Fig. S7

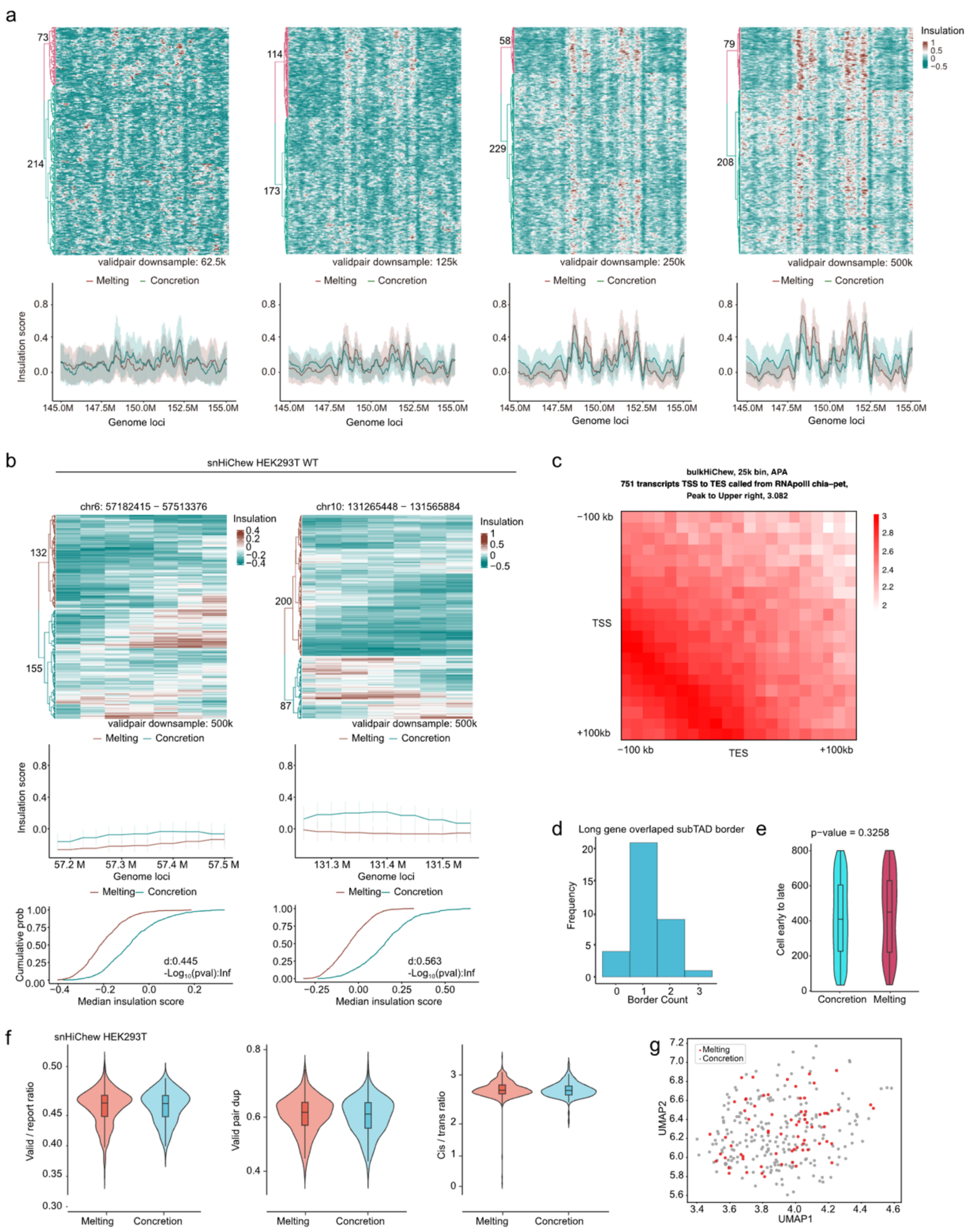
a
validpair downsample: 62.5k
validpair downsample: 125k
validpair downsample: 250k
validpair downsample: 500k
Melting
Concretion
Insulation
Insulation score
Genome loci
b
snHiChew HEK293T WT
chr6: 57182415 ~ 57513376
chr10: 131265448 ~ 131565884
Median insulation score
Cumulative prob
d:0.445
d:0.563
c
bulkHiChew, 25k bin, APA
751 transcripts TSS to TES called from RNApolII chia-pet,
Peak to Upper right, 3.082
TSS
TES
d
Long gene overlaped subTAD border
Frequency
Border Count
e
p-value = 0.3258
Cell early to late
f
snHiChew HEK293T
Valid / report ratio
Valid pair dup
Cis / trans ratio
g
UMAP1
UMAP2

Fig. S7: Chromatin organization analysis in HEK293T Cells. **a**, Evaluation of chromatin organization across sequencing depth intervals of 62.5k, 125k, 250k, and 500k valid pairs per cell. Contact maps and insulation score patterns are presented, with upper panels featuring insulation score heatmaps categorized by melting (red) and concretion (green) states. Lower panels illustrate the corresponding insulation score distributions, highlighting the correlation between sequencing depth and chromatin state resolution. **b**, Assessment of chromatin organization in wild-type HEK293T cells within specific genomic loci. Analysis of chr6:57.2M-57.5M and chr10:131.3M-131.5M regions reveals distinct melting and concretion state patterns. Insulation score distributions are presented for both states, accompanied by cumulative distribution analyses yielding KS test statistics (d values: 0.445 and 0.563). **c**, APA plot displaying polymerase-based chromatin interactions between TSS and TES (751 genes), with color intensity representing interaction frequency (darker red indicating stronger interactions). **d**, Histogram showing the frequency distribution of long genes that overlap subTAD borders, with the x-axis representing border count and y-axis showing frequency. The analysis demonstrates how often long genes span across topological domain boundaries. **e**, Violin plot comparing cell array distance to the nuclear lamina between concretion (blue) and melting (red) chromatin states. The *p*-value of 0.3258 indicates the statistical significance of the observed differences in nuclear peripheral positioning between the two chromatin conformational states. **f**, Comparative quality control assessment of snHiChew HEK293T cells categorized by chromatin state. Violin plots illustrate the distribution of three critical performance metrics across melting (red) and concretion (blue) cell populations: valid report ratio (left), valid pair duplication ratio (middle), and cis-trans ratio (right). Two-sided Wilcoxon signed-rank test was used. **g**, UMAP embedding of snHiChew HEK293T single-cell chromatin contact matrices, revealing two distinct chromatin organizational states. Cells are colored by their chromatin state classification: melting state and concretion state.

Fig. S8

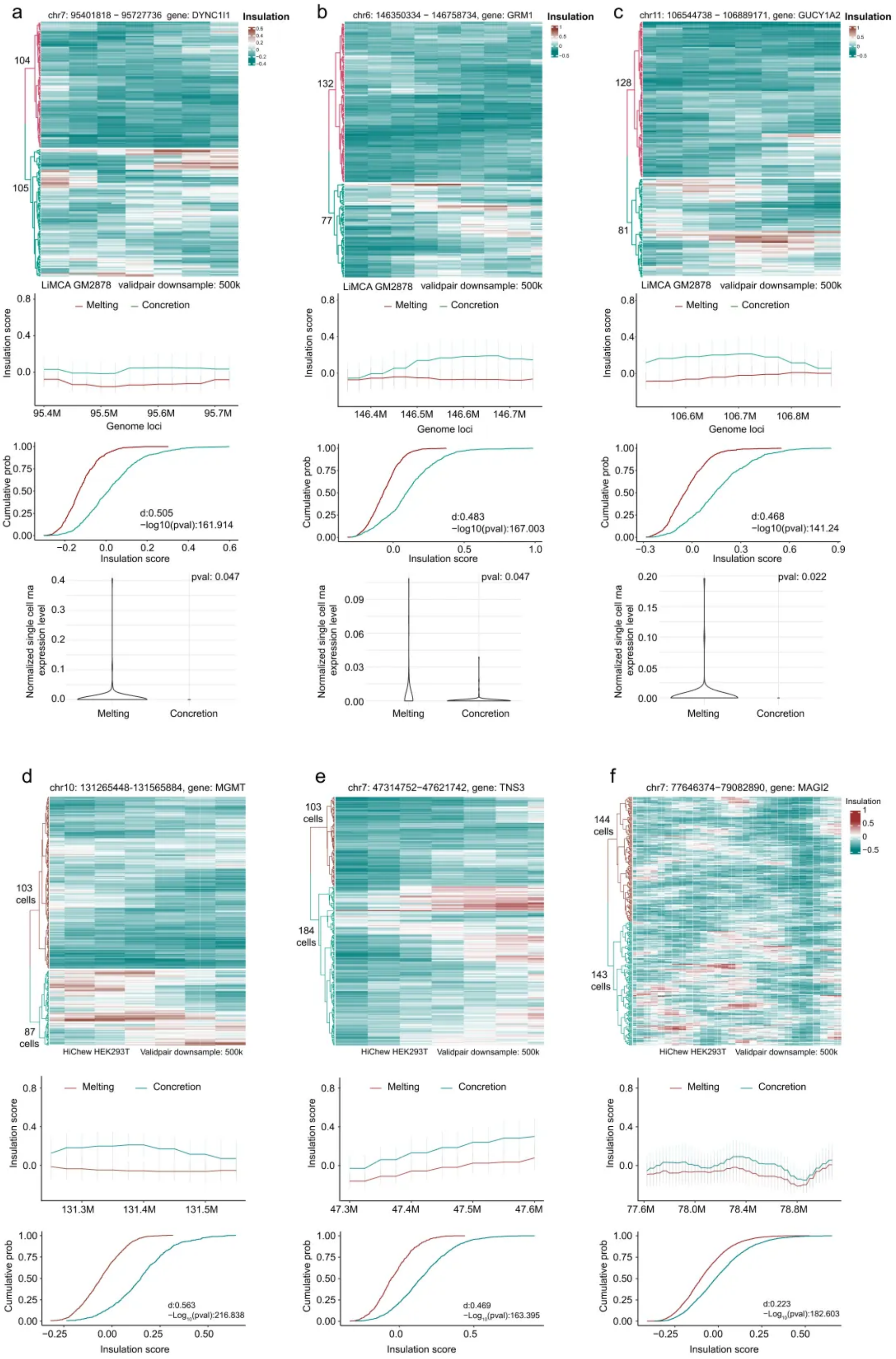

a
chr7: 95401818 − 95727736 gene: DYNC1I1
Insulation
104
105
LiMCA GM2878 validpair downsample: 500k
Melting
Concretion
Insulation score
Genome loci
Cumulative prob
d:0.505
−log10(pval):161.914
Insulation score
pval: 0.047
Normalized single cell rna expression level
b
chr6: 146350334 − 146758734, gene: GRM1
132
77
d:0.483
−log10(pval):167.003
c
chr11: 106544738 − 106889171, gene: GUCY1A2
128
81
d:0.468
−log10(pval):141.24
pval: 0.022
d
chr10: 131265448-131565884, gene: MGMT
103 cells
87 cells
HiChew HEK293T Validpair downsample: 500k
d:0.563
e
chr7: 47314752−47621742, gene: TNS3
103 cells
184 cells
d:0.469
f
chr7: 77646374−79082890, gene: MAGI2
144 cells
143 cells
d:0.223

Fig. S8 Chromatin state analysis of specific gene regions in GM12878 cells in LiMCA. a, Heatmap of insulation scores across the DYNC1I1 gene region (chr7: 95401818 - 95727736) in GM12878 cells. Top panel shows hierarchical clustering of 104 cells based on insulation score patterns, with melting (red) and concretion (teal) chromatin states clearly distinguishable. Middle panel displays mean insulation score profiles for melting (red line) and concretion (teal line) states across the genomic region. Bottom panels show cumulative probability distributions of insulation scores with KS test maximum distance (d=0.505) and associated *p*-value (0.047), followed by normalized RNA expression level comparison between melting and concretion states for this gene region. **b**, Similar analysis for the GRM1 gene region (chr6: 146350334 - 146758734) showing 132 cells hierarchically clustered by insulation score. The cumulative probability distribution shows a KS test maximum distance of d=0.483 with p-value of 0.047, indicating significant differences in chromatin organization between melting and concretion states. RNA expression analysis demonstrates higher expression levels in the melting state compared to concretion. **c**, Analysis of the GUCYIA2 gene region (chr11: 106541738 - 106889171) showing 128 cells with distinct insulation score patterns between melting and concretion states. The cumulative probability distribution indicates a KS test maximum distance of d=0.468 with *p*-value of 0.022, further supporting the relationship between chromatin state and gene regulation. Two-sided Wilcoxon signed-rank test was used. The expression analysis shows higher transcriptional activity in the melting state compared to the concretion state. All analyses were performed using LiMCA GM12878 cells with 500k valid pairs per cell downsampling. The results consistently demonstrate the relationship between chromatin organization states (melting vs. concretion) and transcriptional activity across multiple genomic loci, supporting a functional link between chromatin structure and gene expression. **d**, Heatmap of insulation scores across the MGMT gene region (chr10: 131265448-131565884) in HEK293T cells. **e-f**, Similar analysis for the TNS3 gene region (chr7: 47314752−47621742) and MAGI2 gene region (chr7: 77646374−79082890).

Fig. S9

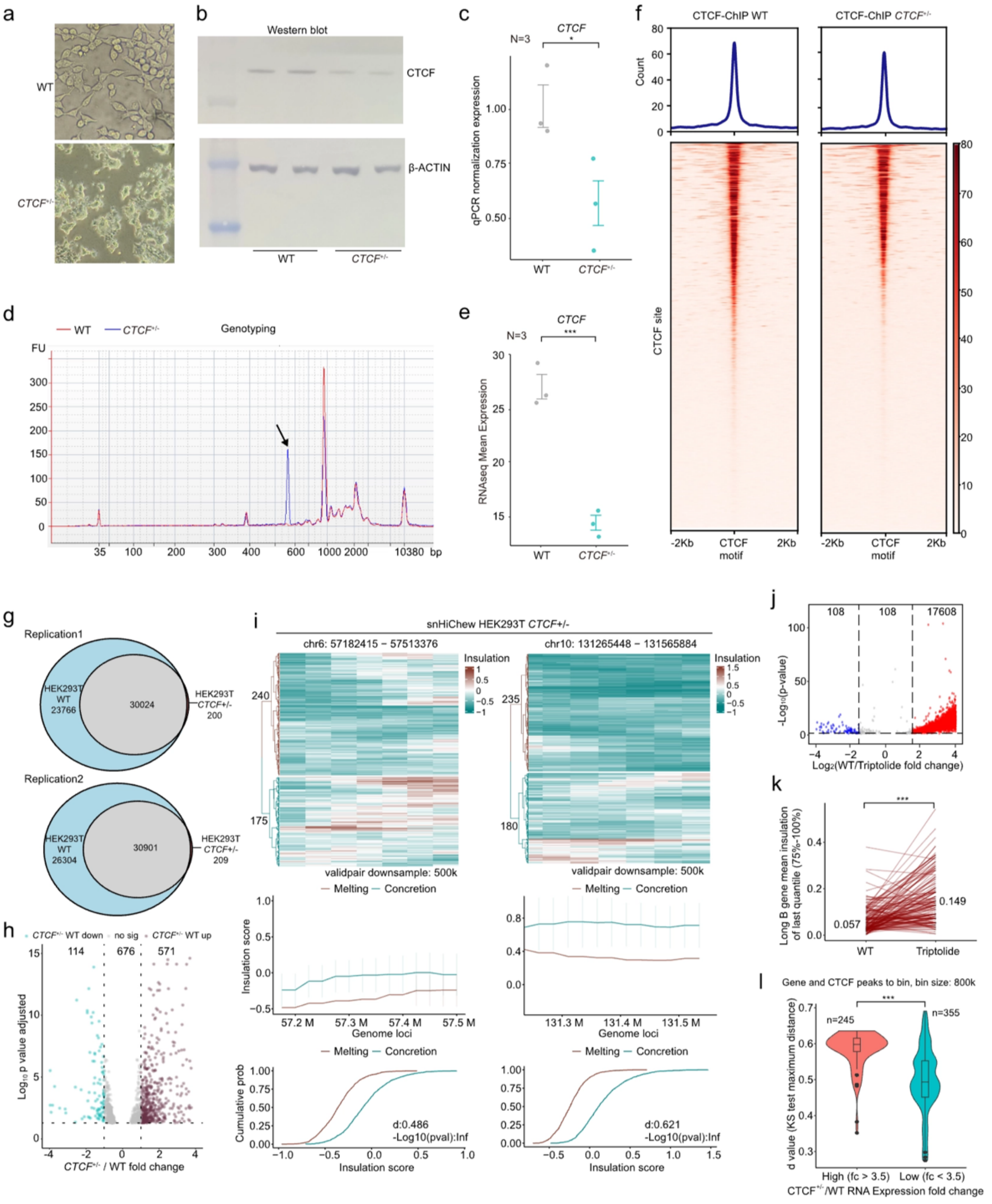

a
WT
CTCF+/-
b
Western blot
CTCF
β-ACTIN
WT
CTCF+/-
c
CTCF
N=3
qPCR normalization expression
WT
CTCF+/-
d
WT
CTCF+/-
Genotyping
FU
bp
e
CTCF
N=3
RNAseq Mean Expression
f
CTCF-ChIP WT
CTCF-ChIP CTCF+/-
Count
CTCF site
-2Kb
CTCF motif
2Kb
g
Replication1
HEK293T WT 23766
30024
HEK293T CTCF+/- 200
Replication2
HEK293T WT 26304
30901
HEK293T CTCF+/- 209
h
CTCF+/- WT down
no sig
CTCF+/- WT up
114
676
571
Log10 p value adjusted
CTCF+/- / WT fold change
i
snHiChew HEK293T CTCF+/-
chr6: 57182415 – 57513376
chr10: 131265448 – 131565884
Insulation
validpair downsample: 500k
Melting
Concretion
Insulation score
Genome loci
Cumulative prob
d:0.486
-Log10(pval):Inf
d:0.621
-Log10(pval):Inf
j
108
108
17608
-Log10(p-value)
Log2(WT/Triptolide fold change)
k
Long B gene mean insulation of last quantile (75%-100%)
0.057
0.149
WT
Triptolide
l
Gene and CTCF peaks to bin, bin size: 800k
n=245
n=355
d value (KS test maximum distance)
High (fc > 3.5)
Low (fc < 3.5)
CTCF+/-/WT RNA Expression fold change

Fig. S9: *CTCF* knockdown validation and analysis of altered in 3D genome, epigenome, and transcription. a, Comparative bright-field microscopy analysis reveals distinct morphological variations between wild-type HEK293T cells and *CTCF*$^{+/-}$cells. **b**, Protein expression analysis through Western blot, utilizing β-ACTIN as an internal control, validates reduced *CTCF* protein levels in knockdown cells. **c**, Quantitative assessment via RT-qPCR confirms diminished *CTCF* expression in *CTCF*$^{+/-}$cells relative to wild-type controls (N=3, *p*-value = 0.0348). The error bar represents standard error (SE). Significance was assessed using two-sided two-sample t-test. **d**, Molecular characterization through fragment length distribution analysis demonstrates differential patterns between wild-type (red) and *CTCF*$^{+/-}$(blue) cells post-genotyping PCR, with the anticipated fragment size variation indicated. **e**, RNA sequencing analysis quantifies *CTCF* expression differences between wild-type and *CTCF*$^{+/-}$cells (N=3, *p*-value = 0.0017). The error bar represents the standard error (SE). Significance was assessed using two-tailed two-sample t-test. **f**, *CTCF* ChIP-seq fragment count spanning *CTCF* motifs. The motif center at 0 on the *x*-axis indicates the *CTCF* motif position. The *y*-axis indicates the ChIP-seq fragment count among all the molecules across *CTCF* motifs. The fragment count heatmaps around *CTCF* sites are shown at the bottom. Each row represents a genomic region with a *CTCF* site. **g**, Comparative ChIP-seq analysis visualized through Venn diagrams illustrates the distribution of *CTCF* binding sites between wild-type and knockdown conditions across two biological replicates (Rep1 and Rep2), with numerical representation of shared and unique peaks. **h**, Differential gene expression analysis presented as a volcano plot illustrates transcriptional changes between wild-type and *CTCF*$^{+/-}$cells, with significant alterations (fold change > 2, p < 0.05) denoted in blue (downregulated) and red (upregulated). **i**, Investigation of *CTCF*-knockdown effects on chromatin organization. Analyses of chr6:57.2M-57.5M and chr10:131.3M-131.5M regions in *CTCF*$^{+/-}$cells demonstrate modified chromatin architecture. Insulation score distributions and cumulative analyses (d values: 0.486 and 0.621) indicate significant alterations in chromatin organization following *CTCF* depletion. **j**, Volcano plot showing differential RNA analysis between wild-type and triptolide-treated conditions. The plot displays -Log10(FDR) versus Log2(Triptolide/WT fold change), with significant changes highlighted in blue (downregulated) and red (upregulated). The numbers above indicate the count of differentially bound sites in each category. **k**, Pairwise evaluation of genome-wide long B compartment genes (n=752) melting level (mean insulation score of last quantile) differences between wild-type and triptolide treated samples (contacts>250K). Same gene of the two samples is connected with a line. Statistical significance is labeled. A two-sided Wilcoxon signed-rank test was used. **l**, In *CTCF*$^{+/-}$cells, genes with high fold changes (*CTCF*/WT>3) exhibit greater KS maximum distances than genes with lower fold changes.

Fig. S10

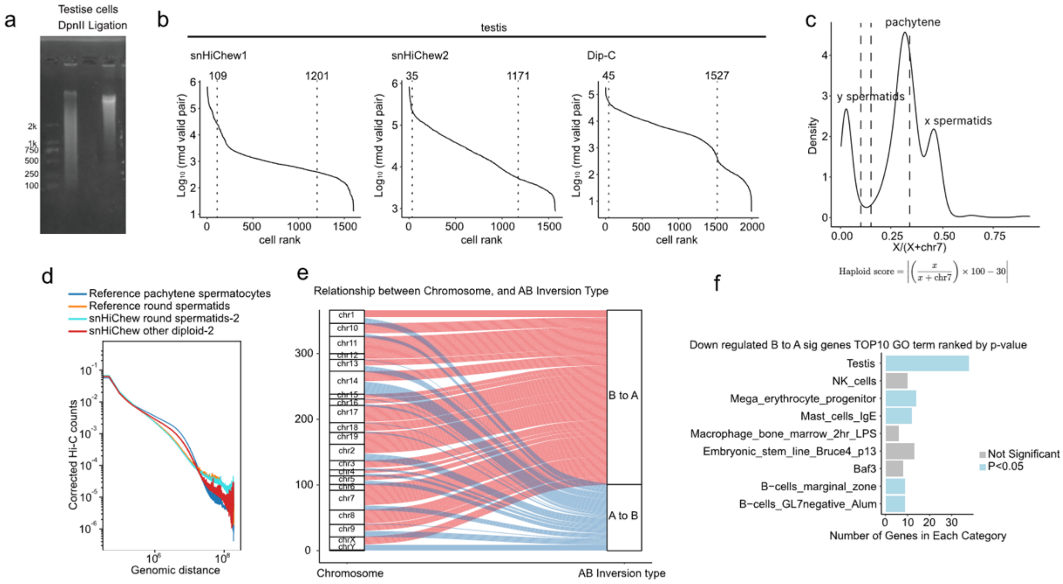

$$\text{Haploid score} = \left|\left(\frac{x}{x+\text{chr7}}\right)\times 100 - 30\right|$$

Fig. S10: Chromatin organization analysis in testis samples. a, Evaluation of testis cell digestion efficiency through gel electrophoresis analysis. The results reveal DNA fragmentation patterns following DpnII digestion and ligation, indicating reduced digestion efficiency in testis samples relative to cultured cells. **b**, Systematic assessment of valid pair distribution in testis cells. The analysis encompasses 1200 cells post-doublet removal, yielding ~1100 cells suitable for analysis. **c**, Cellular differentiation analysis utilizing X chromosome content quantification. This methodological approach enables distinction between spermatocyte/spermiogonia and round spermatids through haploid score determination. **d**, Comparative analysis of quality control parameters across experimental conditions in testis samples and other samples. **e**, Sankey diagram illustrating the relationship between chromosomes and AB compartment inversion types in testis cells. Each chromosome (chr1 through chrX) is connected by flow lines to demonstrate the genomic distribution of compartment switches, with B-to-A transitions (indicating chromatin activation) and A-to-B transitions (indicating chromatin repression) shown separately. The width of connecting lines represents the relative frequency of inversions on each chromosome, revealing chromosome-specific patterns of compartment reorganization during spermatogenesis. **f**, Gene Ontology (GO) term enrichment analysis of upregulated genes undergoing B-to-A compartment transitions, ranked by statistical significance (*p*-value).

Fig. S11

a

HEK293T-WT

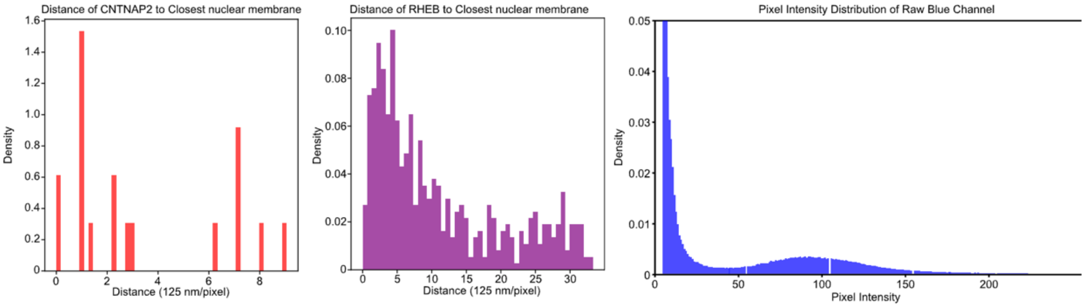

b

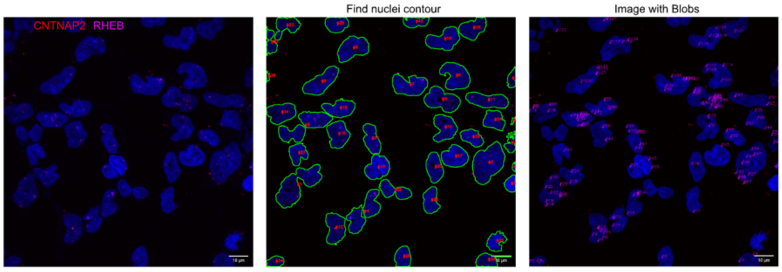

Fig. S11: Analysis of nuclear localization in HEK293T cells. a, Quantitative analysis of the nuclear organization of HEK293T-WT cells showing three key measurements: distance distribution of *CNTNAP2* to the closest nuclear membrane (left, red histogram), distance distribution of RHEB to the closest nuclear membrane (middle, purple histogram), and pixel intensity distribution of raw blue channel (right, blue curve) to remove the background DAPI signal before nuclei contour identification. b, Representative fluorescence microscopy images of HEK293T-WT nuclei showing original DAPI staining (left), computational blob detection and boundary analysis (middle, green outlines), and merged image with detected blobs (right).
