# Supplementary material for: Highly efficient chromatin conformation capture with post-enrichment in single cells by HiChew
Source: Genome Biol. 2026 Apr 27;27:127. doi: 10.1186/s13059-026-04059-1 (PMC13112749; doi:10.1186/s13059-026-04059-1)
Supplement: Supplementary file 9 — Additional file 9: Table S7. Metadata of the CTCF ChIP-seq for HEK293T WT and CTCF knockdown cell library. [file 13059_2026_4059_MOESM9_ESM.pdf]

method supplement note

Evaluation of factors influencing snHiChew/HiChew efficiency

To ensure HiChew/snHiChew can be reliably reproduced in other laboratories, we conducted a comprehensive analysis of factors influencing capture efficiency in our protocol. Our investigation focused on several key parameters: digestion efficiency, antibody enrichment, fragment size, library construction methods, PCR amplification cycles, and alternative enzymes. Digestion efficiency emerged as a critical factor for valid pair ratio because singleton GATC motifs in the middle—which comprise approximately 30% of singletons (Extended Data Fig. 5a left)—are significant byproducts of inefficient digestion. To assess this, we performed a time-course experiment for in situ digestion of the cellular genome. The results revealed a positive correlation between digestion duration and valid pair ratio. Notably, 16h digestion resulted in a significant 40% improvement in the valid pair ratio, highlighting the importance of optimizing digestion conditions in our protocol (Extended Data Fig. 5a middle and right).

Our in vitro experiments demonstrated that Dam-labeled DNA fragments could be fully digested by the methylation-specific enzyme DpnI, suggesting Dam labeling efficiency approaches complete labeling (Extended Data Fig. 5b). Subsequently, we investigated antibody specificity as another factor influencing capture efficiency. Comparative analysis of antibodies from two manufacturers revealed that the Millipore antibody exhibited superior performance (Extended Data Fig. 5c). We also evaluated a recently published stringent SDS buffer protocol, which did not yield significant improvements for bulk samples. Additionally, we explored a two-round immunoprecipitation approach, resulting in a modest ~8% efficiency increase (Extended Data Fig. 5d). However, we determined that this marginal improvement did not justify the additional procedural complexity.

After optimizing the immunoprecipitation (IP) conditions, we quantified the enrichment efficiency using GATC percentage analysis. The results indicated an 8-fold enrichment compared to the pre-IP product (Extended Data Fig. 5e). Notably, GC content analysis of post-IP reads showed comparable coverage to pre-IP samples, suggesting minimal sequence bias introduced by the immunoprecipitation process (Extended Data Fig. 5f).

Our investigation into fragment size effects revealed that longer fragments (500bp) demonstrated a slight increase in enrichment efficiency compared to shorter ones (400bp), with a modest ~3% improvement (Extended Data Fig. 5g). This observation suggests that the Immunoprecipitation (IP) process may introduce a minor bias favoring larger fragments. This hypothesis is further supported by a slight 35bp increase in average fragment length post-IP (Extended Data Fig. 5h,i).

Our evaluation encompassed diverse in vitro library construction methodologies, specifically examining Tn5-based, conventional Y adapter ligation-based, and MDA-based approaches. The analysis indicated superior valid pair ratios when utilizing our ligation-based methods during in vitro experimentation (Extended Data Fig. 5j). Of particular significance, the single-cell Tn5-based method (in vivo) exhibited remarkable performance, achieving a valid pair ratio surpassing 70%. This significant finding merits comprehensive investigation in subsequent discussion.

We subsequently assessed the influence of PCR (before immunoprecipitation) on capture efficiency and potential artifact generation (Extended Data Fig. 5k). Our analysis indicated that increasing PCR cycles led to a slight increase in PCR duplicates. Importantly, we noted a 10% decrease in the valid pair ratio when utilizing higher PCR cycles. We hypothesize that this reduction may be due to PCR bias, potentially resulting in the loss of valid pairs during amplification. However, our investigation into the correlation between PCR cycles and artifact introduction did not produce definitive evidence. Notably, our analysis demonstrated that HiChew actually decreased artifacts by 25% compared to Dip-C, which can be attributed to the enhanced specificity of valid pairs resulting from motif enrichment.

To further showcase HiChew's versatility, we assessed an alternative enzymatic setup. We utilized AluI for genomic digestion and AluI methyltransferase for ligation scar labeling (Extended Data Fig. 5l). This approach yielded a valid pair ratio of approximately 0.43, which is comparable to the Hi-C based method. The high correlation between AluI and Hi-C batches (0.9 in eigenvalue and 0.86 in insulation scores) reinforces the robustness and reproducibility of the HiChew technology across different enzyme configurations.

We evaluated factors influencing snHiChew/HiChew protocol efficiency, including digestion time, antibody specificity, fragment size, and library construction methods. Key findings highlight the protocol's adaptability across

various conditions, with overnight digestion, longer DNA fragments, and Tn5-based library construction showing notable improvements. The study demonstrates HiChew's versatility and robustness, reinforcing its potential as a valuable tool in chromatin conformation capture research.

**Metric**	**HiChew / snHiChew**	**Dip-C (Unenriched)**	**snHi-C (Biotin-Enriched)**
**Valid Pair Ratio** (Bulk)	40-50%	8-20%	~95% (Conventional Hi-C)
**Valid Pair Ratio** (Single-cell)	45-50%	12-20%	>75% (but high duplication)
**Library Complexity**	High (Comparable to Dip-C)	High (but requires massive sequencing)	Low (saturates quickly)
**Key Feature**	**Post-PCR Methylation-based Enrichment**	Whole-genome sequencing (no enrichment)	**Pre-PCR Biotin-based Enrichment**
**Typical Cell Input (Bulk)**	50 - 5,000 cells	50 - 5,000 cells	Millions of cells
**Resolution (with 4M reads/cell)**	High coverage: ~80% bin coverage at 5 kb	Lower coverage: ~70% bin coverage at 5 kb	Low coverage: ~10% bin coverage at 5 kb
**Data Quality (Correlation with Gold-Standard Hi-C)**	High (Eigenvalue: ~0.87, Insulation: ~0.86)	Lower (Eigenvalue: ~0.87, Insulation: ~0.71)	High (but limited complexity)
